# Circulating microRNAs are promising novel biomarkers for drug-resistant epilepsy

**DOI:** 10.1038/srep10201

**Published:** 2015-05-18

**Authors:** Jun Wang, Lan Tan, Lin Tan, Yan Tian, Jing Ma, Chen-Chen Tan, Hui-Fu Wang, Ying Liu, Meng-Shan Tan, Teng Jiang, Jin-Tai Yu

**Affiliations:** 1 Department of Neurology, Qingdao Municipal Hospital, School of Medicine, Qingdao University, Qingdao, China; 2 Department of Neurology, Qingdao Municipal Hospital, College of Medicine and Pharmaceutics, Ocean University of China, Qingdao, China; 3 Department of Neurology, Qingdao Municipal Hospital, Nanjing Medical University, Qingdao, China; 4 Department of Neurology, Qingdao Municipal Hospital, Dalian Medical University, China; 5 Department of Neurology, Nanjing First Hospital, Nanjing Medical University, Qingdao, China

## Abstract

MicroRNAs (miRNAs) open up a new field for molecular diagnosis for cancer and other diseases based on their stability in serum. However, the role of circulating miRNAs in plasma/serum in epilepsy diagnosis is still unclear. The aim of this study was to evaluate whether miRNAs can be used as biomarkers for drug-resistant epilepsy. We measured the differences in serum miRNA levels between 30 drug-resistant patients and 30 drug-responsive epilepsy patients in discovery and training phases using Illumina HiSeq2000 sequencing followed by quantitative reverse transcriptase polymerase chain reaction (qRT-PCR) assays. The selected miRNAs were then validated in 77 drug-resistant epilepsy patients, 81 drug-responsive epilepsy patients and 85 healthy controls by qRT-PCR. We found that circulating miRNAs are differentially expressed between drug-resistant group and drug-responsive group. MiR-194-5p, -301a-3p, -30b-5p, -342-5p and -4446-3p were significantly deregulated in drug-resistant group compared to drug-responsive group and control group. Among these 5 miRNAs, miR-301a-3p had the best diagnostic value for drug-resistant epilepsy with 80.5% sensitivity and 81.2% specificity, and was negatively associated with seizure severity. These provide the rationale for further confirmation studies in larger prospective cohorts and in other ethnics.

Epilepsy is estimated to affect about 65 million individuals worldwide^1^. Although the prognosis for the majority of patients is good, up to 30 percent, with drug-resistant epilepsy, do not have remission despite appropriate therapy with antiepileptic drugs(AEDs)^1^. The long-term use of drugs would result in substantial deleterious effects on individual health and quality of life and a heavy burden on society^2^. Therefore, it is significant to distinguish drug-resistant epilepsy with drug-responsive epilepsy early in the course of disease. To date, the early identification is mainly based on clinical manifestations, such as the numbers of seizures before therapy and the response to initial treatment with antiepileptic drugs^3^. However, these characteristics are indefinite and subjective. Thus, definite, objective and noninvasive biomarkers are in need.

Recently, microRNAs (miRNAs) have been proposed as potential diagnostic tools for many diseases due to their characteristics of stability in serum^4^, economical, rapid and noninvasive. Notably, circulating miRNAs have been reported as promising biomarkers with great accuracy for aging^5^, cancer^4^,^6^ and neurodegenerative disorders, such as Parkinson’s disease^7^, multiple sclerosis^8^, Alzheimer’s disease^9^, *et al.* Moreover, several target studies and genome-wide miRNA expression profiling studies have demonstrated that miRNAs were differentially expressed in epilepsy^10^,^11^,^12^,^13^,^14^,^15^,^16^,^17^; some functional investigations have indicated that miRNAs may be implicated in epilepsy by regulating inflammatory response, neuronal apoptosis and transcription factors involved in differentiation^11^,^18^,^19^. But almost all of the studies were based on samples of human brain tissue or animal models.

In present study, we first intended to identify serum-based miRNA biomarkers for detection of drug-resistant epilepsy patients from drug-responsive epilepsy patients. Moreover, we also investigate the relationship between biomarkers and clinical characteristics (e.g. seizure severity, frequency and disease duration).

## Results

### Characteristics of individuals

A total of 303 participants (including 30 patients with drug-resistant epilepsy and 30 patients with drug-responsive epilepsy in discovery and training phases, 77 drug-resistant and 81 drug-responsive patients and 85 healthy controls in validation phase) were recruited to this study. No significant differences of age, gender or Body Mass Index (BMI) were found in discovery and training set (P = 0.155, 0.797, 0.487, respectively), or in validation set (P = 0.114, 0.901, 0.067, respectively). The duration of seizures in patients with drug-resistant epilepsy (ranging from 2 to 32 years in discovery and training phases, from 2 to 39 years in validation phase) was significantly longer than that in patients with drug-responsive epilepsy (ranging from 1 to 30 years in discovery and training phases, from 1 to 20 years in validation phase) (P < 0.001). The detailed clinical characteristics of individuals were listed in Table 1.

### Distinct circulating miRNA profilings of drug-resistant epilepsy vs drug-responsive epilepsy in discovery set

**Figure 1:**
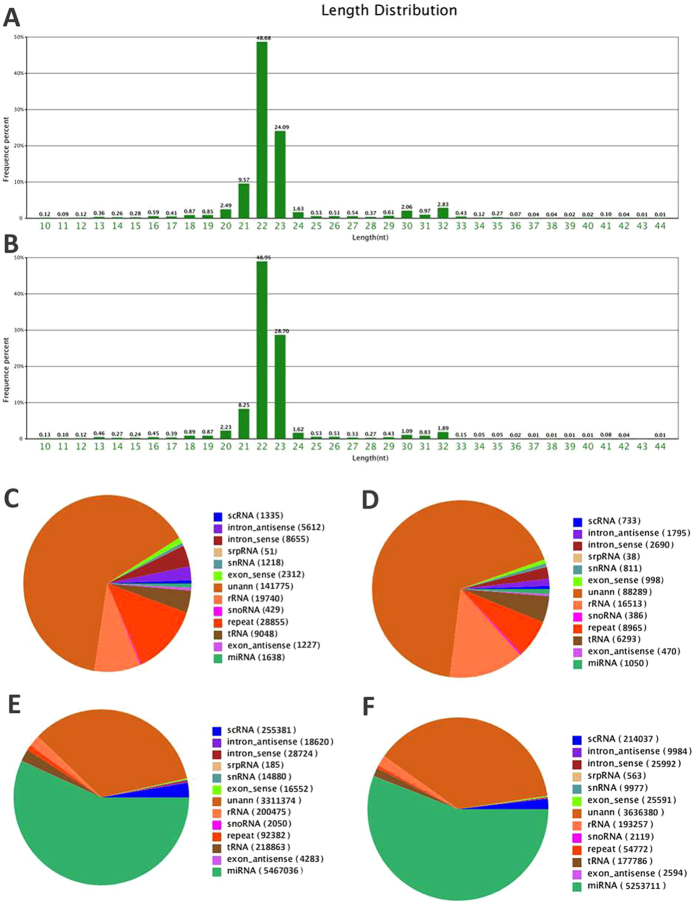
The circulating miRNAs signatures identified by Illumina Hiseq2000 sequencing. The length distribution and frequency percentages of the sequences identified in drug-resistant epilepsy samples (**A**) and drug-responsive epilepsy samples (**B**) RNA species in drug-resistant epilepsy samples (**C**) and drug-responsive epilepsy samples (**D**) and RNA read counts in drug-resistant epilepsy samples (**E**) and drug-responsive epilepsy samples (**F**).

In total, genome-wide sequencing identified 10,000,000 raw reads in both drug-resistant group and drug-responsive group. As is shown in Fig. 1A,B, the dominant small RNAs were 22-23nt in length, accounting for 72.77% and 77.65% of the total reads in drug-resistant and drug-responsive group, respectively. After getting rid of low-quality sequences, sequences shorter than 18 nucleotides, and single-read sequences, 9,630,805 (96.65%) clean reads in drug-resistant group and 9,606,969 (96.40%) clean reads in drug-responsive group were remained for further analysis. Among these clean reads, 6192151 (64.3%) reads in drug-resistant group and 5983857 (62.29%) reads in drug-responsive group were perfectly mapped to the human genome in Genbank. Although miRNAs accounted only a tiny fraction of the total small RNAs, the expression levels of individual miRNAs were relatively high. Moreover, both the number of the unique miRNA sequences and the amount of miRNA species were mildly higher in drug-resistant epilepsy patients compared with drug-responsive epilepsy patients (1638vs 1050, 5467036 vs 5253711, respectively) (Fig. 1C–F). The deep sequencing data and analyses of differentially expressed miRNAs were listed in Supplementary Table S1. Genome-wide sequencing showed that 185 miRNAs were differentially expressed between drug-resistant group and drug-responsive group. The miRNA levels were considered to be significantly different only if they met the following criteria^20^: (1) having at least 10 copies in drug-resistant or drug-responsive groups; (2) showing a fold-change (log_2_drug-resistant/drug-responsive) >2 or <−2 between each comparisons (P < 0.05). According to these criteria, we found that 12 miRNAs were downregulated (miR-194-5p, -204-5p, -221-5p, -301a-3p, -30b-5p, -342-5p, -3605-5p, -4446-3p, -598-3p, -874-3p, -889-3p and novel-mir-451) and 3 were upregulated (miR-574-5p, novel-mir-67 and novel-mir-9) in drug-resistant group compared to drug-responsive group (**Supplementary Table S1**). Among the 15 deregulated miRNAs, 3 miRNAs (novel-mir-451, -67 and -9) were not listed in miRBase (Release 21, http://www.mirbase.org/) and seems to be novel miRNAs.

### Investigation of 15 selected single miRNAs using qRT-PCR in training set

**Figure 2:**
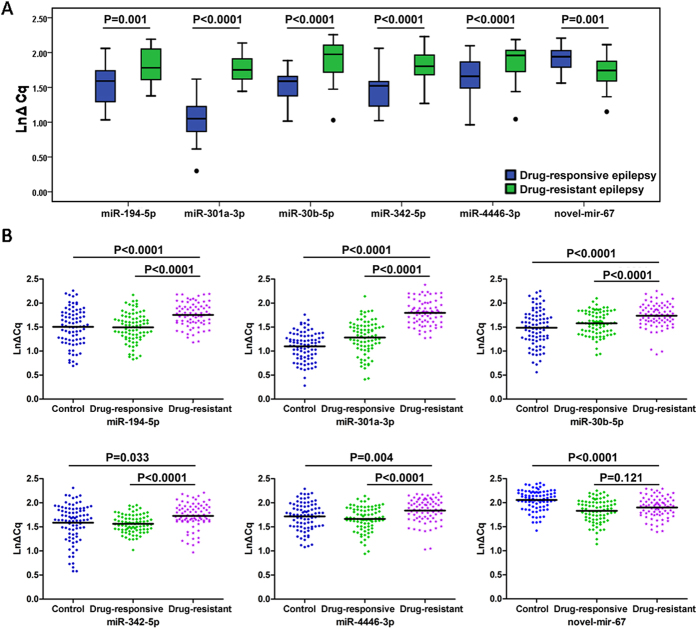
Differential expression of miRNAs in drug-resistant epilepsy patients. **A** Significantly downregulated miRNAs in drug-resistant epilepsy compared to drug-responsive epilepsy in secondary screening stage. **B** Large-scale validation of the 6 miRNAs selected from secondary screening in drug-resistant epilepsy compared to drug-responsive epilepsy and controls. The blue dots represent health control group, green dots represent drug-responsive group, and purple dots represent drug-resistant group. Expression levels of the miRNAs (LnΔCq scale at Y-axis) were normalized to spiked-in cel-miR-39. The line represents the median value. Mann-Whitney U test was used to determine statistical significance.

The expression levels of the 15 miRNAs selected by high-throughput sequencing were determined using qRT-PCR in a cohort of 30 drug-resistant patients and 30 drug-responsive patients (**Supplementary Table S2**). The primers for real-time PCR of each miRNA were listed in **Supplementary Table S3**. MiRNA levels were normalized to cel-miR-39. All samples were measured in triplicates and the mean values were used for analysis. Only miRNAs with a Cq value <36, a detection rate >75% in both groups, and a p value <0.05 were selected for further analyses^20^. As a result, miR-194-5p, -301a-3p, -30b-5p, -342-5p and -4446-3p were significantly decreased in drug-resistant patients when compared with drug-responsive patients; while novel-mir-67 was increased in drug-resistant patients (Fig. 2A). The detection rates of miR-3605-5p and novel-mir-9 were less than 75%; miR-221-5p and miR-889-3p displayed poor results of melting curving analysis; no significant difference was observed in the levels of miR-204-5p, -574-5p, -598-3p, -874-3p and novel-mir-451 between drug-resistant patients and drug-responsive patients (P > 0.05).

### Confirmation of 6 identified single miRNAs using qRT-PCR in large-scale validation set

**Figure 3:**
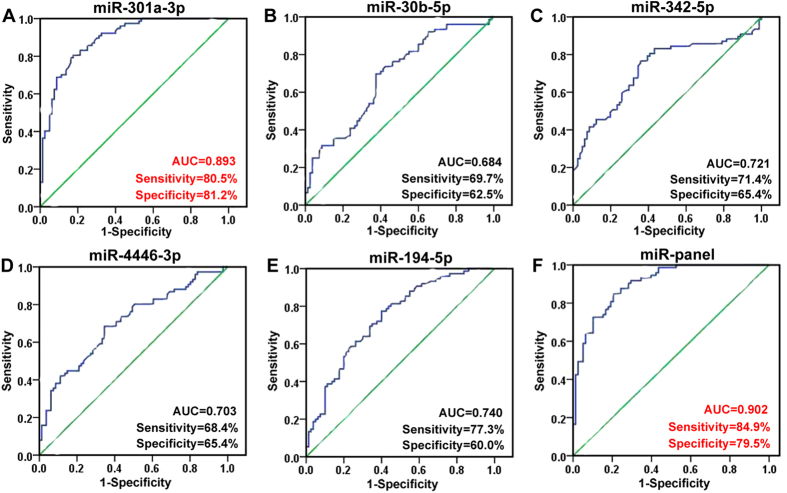
Receiver operating characteristic (ROC) curve analysis using 5 serum miRNAs selected in large-scale validation and the miRNA panel for discriminating drug-resistant epilepsy from drug-responsive epilepsy. AUC, area under the ROC curve.

To further evaluate the diagnostic value of the 6 miRNAs (miR-194-5p, -301a-3p, -30b-5p, -342-5p, -4446-3p and novel-mir-67) identified in the training phase, the expression levels of these 6 miRNAs were measured on a total of 243 serum samples including 77 drug-resistant patients, 81 drug-responsive patients and 85 healthy controls (Supplementary Table S4) . The results revealed that miR-194-5p, -301a-3p, -30b-5p, -342-5p and -4446-3p were significantly downregulated in drug-resistant patients compared with drug-responsive patients and healthy controls (Fig. 2B). Unfortunately, no significant difference was detected in the expression level of novel-mir-67. ROC curve analyses (Fig. 3A–F) indicated that miR-301a-3p was the most valuable biomarker for differentiating drug-resistant epilepsy from drug-responsive epilepsy with an AUC of 0.893 (95%CI: 0.844-0.941). At the cutoff value of 1.5634 for miR-301a-3p, the optimal sensitivity and specificity were 80.5% and 81.2%, respectively (Fig. 3A). Multivariate logistic regression analyses on variables including age, gender and BMI revealed that miR-301a was a potential biomarker for the diagnosis of drug-resistant epilepsy (P = 4.10 × 10^−5^). The odds ratio for cases with expression level of miR-301a-3p less than 1.5634 being associated with drug-resistant epilepsy was 19.486 (95%CI: 8.698-43.655). In addition, we combined these miRNAs to form different panels, and evaluated these panels’ diagnostic value. The miR-194-5p/miR-301a-3p/miR-30b-5p/miR-4446-3p combination (miR-panel) showed better diagnostic value than other combination with a similar AUC of 0.902 to that of miR-301a-3p (95%CI: 0.855-0.948; sensitivity: 84.9%, specificity: 79.5%) (Fig. 3F), indicating a poor additive effect of the 4 miRNAs.

### Relationship between serum levels of miR-301a-3p and clinical characteristics

**Figure 4:**
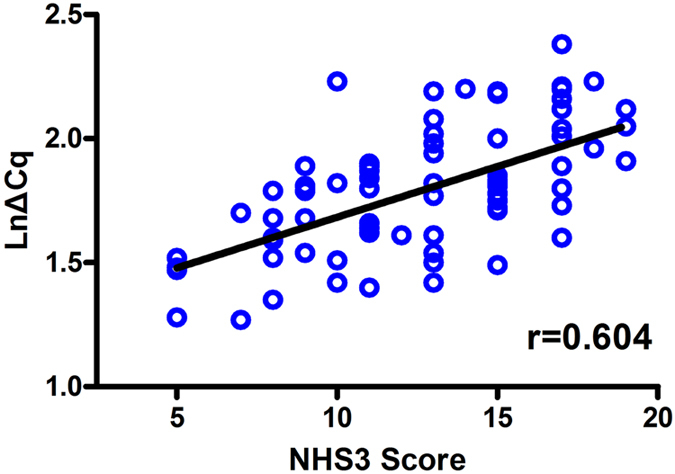
Correlation between the National Hospital Seizure Severity Scale (NHS3) scores and the expression level of miR-301a-3p. Serum miR-301a-3p level was significantly associated with the NHS3 scores in drug-resistant epilepsy patients (r = 0.604, P = 6.2 × 10^−9^). Expression level of miR-301a-3p (LnΔCq scale at Y-axis) were normalized to spiked-in cel-miR-39.

In addition to group comparisons, we performed regression analysis to investigate the association between the expression level of miR-301a-3p with clinical parameters. No significant association was observed between miR-301a-3p and disease duration or seizure frequency (P > 0.05, data not shown). Interestingly, we found that miR-301a-3p level was significantly associated with NHS3 score (r = 0.604, P = 6.2 × 10^−9^) (Fig. 4), indicating that the expression level of miR-301a-3p was negatively associated with seizure severity.

## Discussion

To date, the diagnosis and treatment of drug-resistant epilepsy still suffer from a lack of reliable biomarkers, despite ample efforts have been made^21^,^22^,^23^. While in recent years, miRNAs have gained significant attention and have been proposed as novel biomarkers for the diagnosis of several diseases including some CNS diseases^4^,^6^,^7^,^8^,^9^,^20^ for several reasons. First, miRNAs have been found stable in serum, and the test of miRNAs in blood is broadly accessible, rapid, noninvasive, and economical. Moreover, the development of powerful detection technologies such as high-throughput sequencing has given a significant boost to the search in miRNAs as biomarkers. Over the past 5 years, several target studies and genome-wide miRNA expression profiling studies^10^,^11^,^12^,^14^,^15^,^24^,^25^,^26^ have identified changes to over 100 different miRNAs in epilepsy patients and animal models, particularly in mesial temporal lobe epilepsy (mTLE), about 30% of which are pharmaco-resistant^27^, and provided compelling evidence that epilepsy is associated with widespread changes to miRNA expression.

Here, we provided the first study to identify serum-based miRNA biomarkers for detection of drug-resistant epilepsy from drug-responsive epilepsy. Our results revealed that miRNAs are differentially expressed in serum samples from drug-resistant patients compared with those from drug-responsive patients. In particular, the expression of miR-194-5p, -301a-3p, -30b-5p, -342-5p and -4446-3p were significantly decreased in drug-resistant patients compared to drug-responsive patients and healthy controls. Among these miRNAs, miR-301a-3p had the best diagnostic value for drug-resistant epilepsy and yielded AUC of 0.897 with 80.5% sensitivity and 81.2% specificity in discriminating drug-resistant patients from drug-responsive patients, and was negatively associated with seizure severity.

The miRNA expression profiling and candidate miRNA biomarkers identified in our study showed some overlap, even limited, with previous reports. In 2012, Hu and colleagues^11^ conducted profiling studies using hippocampus from rat model of TLE, and found miR-301a (previous ID of miR-301a-3p) and other 14 miRNAs were down-regulated in TLE rat models. Later, Bot *et al.*^14^ profiled miRNA expression levels in dentate gyri from epileptic rat models and sham operated controls, and found that 57 miRNAs including miR-301a-3p and miR-30b-5p were downregulated, while 9 miRNAs were upregulated in epileptic models. Over the same period, Mckiernan *et al.*^13^ profiled mature miRNA levels in hippocampus from pharmacoresistant TLE patients and controls. Their results showed that 37 miRNAs including miR-301a-3p and miR-30b-5p were significantly downregulated in pharmaco-resistant TLE samples. These results showed that changes to miR-301a-3p and miR-30b-5p were consistent with our findings. However, in the study of Kan and colleagues^12^, which first undertook genome-wide profiling of miRNAs in human epilepsy, miR-301a was upregulated in mTLE patients with and without hippocampal sclerosis in comparison to controls. In addition, a set of miRNAs were deregulated in some studies, but not in others. These differences may be explained by the different standards for the selection of TLE patients or varied criteria for the surgery selection of TLE patients. Moreover, different standards to screen for significantly deregulated miRNAs may also contribute to a difference in results. Additionally, limited sample size, different models and/or brain regions, study design, technical factors, extraneous effects including race, BMI, lifestyle, and other individual characteristics may also influence the profiling of miRNA abundance. These need to be validated in the future.

Although miR-301a-3p has been demonstrated to be deregulated in drug-resistant epilepsy patients in present and previous studies, at this stage, we could not come to the conclusion that miR-301a-3p is ready to be used as a diagnostic biomarker for several limitations. First of all, the sample size in our study, although much larger than previous studies, is still limited. It influences the accuracy of the results. Moreover, participants in our study are from a confined geographic area with less heterogenous background. In addition, miR-301a-3p is also deregulated in some other diseases, including some types of tumors^28^ and Alzheimer’s disease^29^. Therefore, it is not specific for the diagnosis of drug-resistant epilepsy. Last, it is still not clear whether the deregulation of miRNAs is a cause or a consequence of epileptogenesis. So, large-scale prospective cohort studies in different ethnic populations are necessary to verify our findings. Nevertheless, some advantages make our study a reliable rationale for future studies. First, we employed a rigorous approach including a high-throughput sequencing of pooled serum samples followed by multiple qRT-PCR validation sets at the individual level. This approach has been widely utilized to identify a particular disease-specific serum miRNA profile. The high-throughput sequencing could detect the genome-wide miRNA expression, but ignored the individual discrepancies. Thus 2 stages of qRT-PCR were following to verify the different expression levels of selected miRNAs at individual level. Compared to other methods of measuring miRNA expression levels, RT-PCR assay is not affected by genomic DNA contamination, and is a sensitive and accurate method for assessing miRNA expression. Moreover, to make the result of qRT-PCR more accurate, we measured all samples in triplicates and used the mean value for analysis. In addition, our study is specially designed to explore different expression levels of miRNAs between drug-resistant and drug-responsive patients, and we also verified the differences between drug-resistant patients and controls.

The understanding of miRNA expression patterns as potential biomarkers for diagnosis of drug-resistant epilepsy is still in its infancy, and the miRNA targets and the molecular mechanisms concerning how miRNAs regulate epileptogenesis are not fully understood. Individual miRNAs can have several targets within the same cell and impact more than one pathway. In neurons, miRNAs have been found to regulate translation of a wide range of proteins^30^, including proteins involved in neuronal morphology^31^, channels^32^, neuronal migration^33^ among others. A functioning miRNA system is also required in astrocytes with loss of miRNA biogenesis producing neurodegeneration and seizures^34^. In order to obtain a further understanding of the differentially expressed miRNAs in drug-resistant epilepsy, we predicted the potential targets of selected miRNAs using the miRNA target prediction databases—RNAhybrid and miRanda. A network of miRNAs and mRNAs of target genes is presented (**Supplementary Fig.S1**). Within the network, many genes are related to inflammation and apoptosis, such as MAPK1, ATM, MYD88, RBL1, TRAF6, PIK3CD, IFNAR2, etc, indicating that these miRNAs may play a role in drug-resistant epilepsy through inflammation and apoptosis. These pathways may represent interesting novel targets for mechanism investigation and therapeutic interventions. In addition, miR-301a-3p is a potential biomarker in our study. It has been revealed that miR-301a-3p was involved in inflammatory response through impacting NF-κB signaling pathway in cancer^28^. Further research is necessary to explore how miR-301a-3p function in epilepsy, involved in inflammation or some other mechanisms.

In conclusion, we first performed a comprehensive investigation of circulating miRNAs in drug-resistant epilepsy. In this report, we identified 5 serum miRNAs to distinguish drug-resistant epilepsy patients with drug-responsive epilepsy patients and healthy controls. Among these miRNAs, miR-301a-3p has strong potential to discriminate drug-resistant epilepsy from drug-responsive epilepsy with 80.5% sensitivity and 81.2% specificity. Our results contribute to the new avenue of miRNA biology in drug-resistant epilepsy and provide the rationale for larger prospective cohort studies in different ethnic populations, which are certainly needed to further confirm our preliminary results of deregulated serum-based miRNAs.

## Methods

### Study design and patients

**Figure 5:**
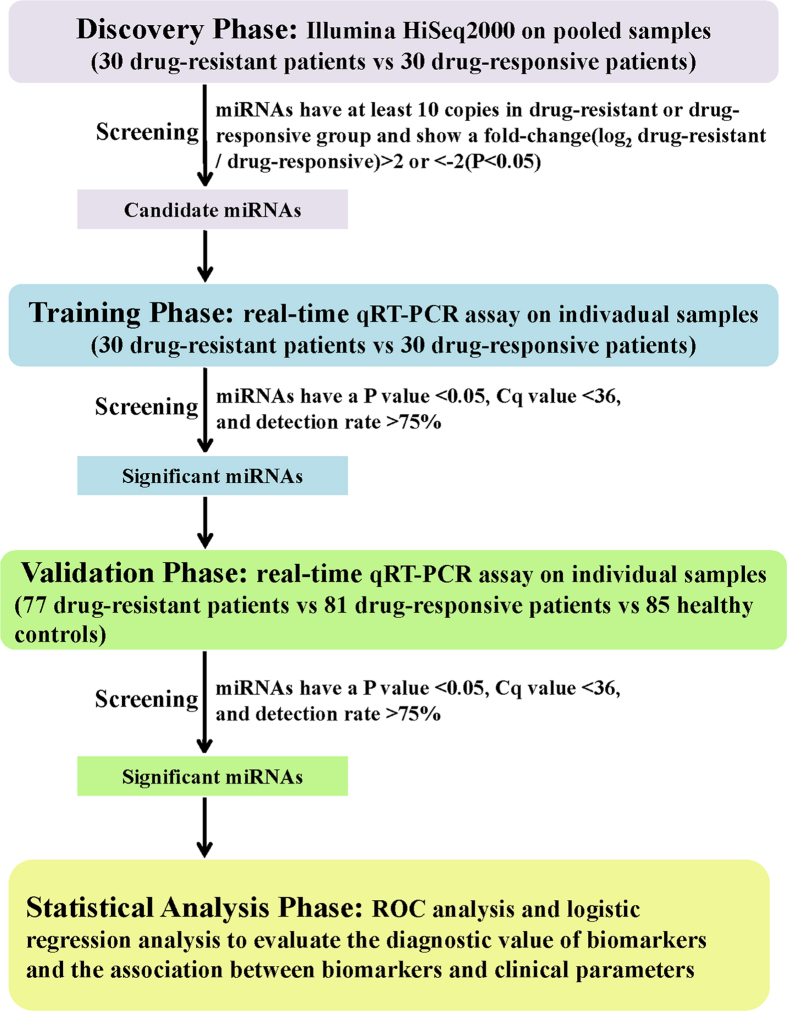
Overview of the study design. qRT-PCR, quantitative reverse transcriptase polymerase chain reaction.

The present study enrolled 107 clinically diagnosed patients with drug-resistant epilepsy, 111 patients with drug-responsive epilepsy and 85 healthy controls matched for age, gender and BMI between October, 2013 and May, 2014. A multiphase case-control study was designed to identify serum miRNAs as biomarkers for drug-resistant epilepsy (Fig. 5). In the discovery phase, we subjected pooled serum samples from 30 drug-resistant patients and 30 drug-responsive patients to Illumina HiSeq 2000 technology to select miRNAs whose expression were altered in drug-resistant patients compared to drug-responsive patients. Subsequently, we refined the number of serum miRNAs included as the drug-resistant signature by a 2-stage experimental procedure using real-time quantitative reverse transcriptase polymerase chain reaction (qRT-PCR) assays. The training phase used serum samples from the 30 drug-resistant patients and 30 drug-responsive patients that had been assessed by Illumina HiSeq 2000 technology, whereas the validation phase used serum samples from additional 77 drug-resistant patients, 81 drug-responsive patients and 85 healthy controls.

All the patients were recruited from the Department of Neurology at Qingdao Municipal Hospital, and several other hospitals in Shandong Province. And all patients went through comprehensive clinical examination, including a medical history, physical and psychiatric examination, laboratory examination, cranial magnetic resonance imaging scans and electroencephalogram. Major exclusion criteria were a history of autoimmune diseases, allergic response, immune deficiency disorder, diabetes, heart disease, stroke, atherosclerosis, psychiatric illness, malignancy, severe cognitive impairment, or a systemic or central nervous system (CNS) infection 2 weeks before sample collection. All patients with drug-resistant epilepsy were evaluated for seizure frequency using seizure diaries and seizure severity using the National Hospital Seizure Severity Scale (NHS3)^35^. The control subjects were recruited from the Health Examination Center of the Qingdao Municipal Hospital, and were confirmed healthy and neurologically normal by medical history, general examinations, laboratory examinations, and have no history of seizures or exposure to AEDs. An informed consent to participate in this study was obtained from each subject, and the study protocol was approved by the Ethics Committee of Qingdao Municipal Hospital. All the experiments described here were in accordance with the guidelines and regulations issued by the Ethics Committee of Qingdao Municipal Hospital.

In this study, epilepsy was diagnosed as idiopathic or cryptogenic epilepsy according to the criteria proposed by the International League Against Epilepsy in 2001^36^. Drug-resistant epilepsy was defined as failure of adequate trails of two tolerated and appropriately chosen and used AED schedules (whether as monotherapies or in combination) to achieve sustained seizure freedom. In our study, all the patients with drug-resistant epilepsy were still on medications at the time of serum testing. Drug-responsive epilepsy was defined as freeing from seizures for the period of at least 12 months^37^.

### Blood processing

Up to 6 ml whole blood was collected from each participant, and was processed for serum isolation within 3 hours of collection by centrifugation at 3,000 r.p.m. for 5 min at room temperature, followed by a 5 min centrifugation at 12,000 xg at 4 °C^20^. The serum samples were stored at −80°C and were not thawed until use. The hemolytic serum samples were excluded.

### Serum small RNA library construction and sequencing

We mixed 300 μl of each serum sample from 30 drug-resistant and 30 drug-responsive patients separately. Total RNA of each mixed serum was isolated using a scaled-up version of the mirVana™ PARIS™ Kit (Ambion, USA) protocol^6^. The final RNA was eluted in 100 μl of preheated (95 °C) elution solution. The concentration and purity of RNA solution were examined by measuring the absorbance at 260-280 nm using the NanoDrop Lite Spectrophotometer (Thermo, Germany). After that, the 18- to 30-nt small RNAs were fractionated, and then were ligated to a 5’ and a 3’ adaptor sequentially. Next, the 5’-, 3’-ligated small RNA solution was reverse-transcribed to cDNA, followed by PCR with primers complementary to the adaptor sequences. Finally, the two generated libraries were sequenced using the Illumina Cluster Station and Genome Analyze (Illumina Inc, CA, USA) at BGI according to the manufacturer’s protocol.

### MiRNA quantification by real-time qRT-PCR

Twenty μl total RNA solution was isolated from 400 μl serum of each sample using the mirVana™ PARIS™ Kit according to the manufacturer’s protocol. To allow for the normalization of sample-to-sample variation in RNA isolation, synthetic *C.* elegans miRNA cel-miR-39^38^,^39^ was added (25 fmol in a 5 μl total volume) to each denatured sample after combining the serum sample with 2×Denaturing Solution^6^. Then the total RNA was reversely transcribed into cDNA in a final volume of 20 μl using One Step PrimeScript miRNA cDNA Synthesis Kit (Takara, Japan). Quantitative real-time PCR was conducted for each sample using SYBR Premix Ex Taq™ II (Takara, Japan) and CFX96 real-time PCR detection system (Bio-rad, Germany) in a final 25 μl reaction volume according to the manufacturer’s protocol. All miRNA primers were purchased from Takara and Tiangen (Beijing, China). At the end of PCR cycles, melting curve analyses were performed to validate the specific generation of the expected PCR products. Each sample was run in triplicates for analysis.

### Statistical analysis

The expression levels of miRNAs for qRT-PCR were normalized to cel-miR-39^40^, and were calculated utilizing the 2^−ΔΔCt^ method^41^. Expression levels of miRNAs were compared using the Kruskall-Wallis test or the Mann-Whitney U test. Receiver Operator Characteristic (ROC) curves and area under the ROC curve (AUC) were established to evaluate the diagnostic value of serum miRNAs for differentiating between drug-resistant group and drug-responsive group. The correlations between the variables were assessed with the Pearson’s correlation coefficient. Clinical characteristics were compared using χ^2^ test of independence for qualitative variables, ANOVA or t-test of quantitative variables with normal distribution, the non-parametric Kruskall-Wallis test or the Mann-Whitney U test of quantitative variables with skewed distribution. A p value of less than 0.05 was considered statistically significant. All analyses were performed by SPSS 17.0 software (SPSS, Chicago, IL, USA) or Graphpad Prism (version 5.0; Graphpad software).

## Additional Information

**How to cite this article**: Wang, J. *et al.* Circulating microRNAs are promising novel biomarkers for drug-resistant epilepsy. *Sci. Rep.* **5**, 10201; doi: 10.1038/srep10201 (2015).

## Supplementary Material

Supporting Information

## Figures and Tables

**Table 1:** Clinical characteristics of individuals.

	**Discovery and training set**			**Validation set**			
	**Drug-resistant**	**Drug-responsive**	**P value**	**Drug-resistant**	**Drug-responsive**	**Control**	**P value**
No.	30	30		77	81	85	
Age, mean ± SD(y)	31.43 ± 8.29	26.80 ± 13.44	0.155	32.44 ± 13.01	29.28 ± 10.86	32.49 ± 9.57	0.114
Female:Male	14:16	16:14	0.797	37:40	40:41	39:46	0.901
BMI, mean ± SD(kg/m^2^)	23.27 ± 3.98	22.31 ± 3.94	0.487	24.20 ± 3.88	22.78 ± 4.31	23.39 ± 3.26	0.067
Disease Duration, median(range)(y)	10(2-32)	2(1-20)	<0.001	11(2-39)	3(1-20)	NA	<0.001
Seizure frequency, median(range)(/6 month)	22(3-127)	NA		12(1-180)	NA	NA	
NHS3 Score	13.23 ± 2.98	NA		11.50 ± 3.30	NA	NA	
							
AED therapy at the last clinic visit							
Monotherapy	2(6.7%)	21(70.0%)		7(9.1%)	62(76.5)	NA	
Polytherapy	28(93.3%)	9(30.0%)		70(90.9%)	19(23.5)	NA	

Abbreviations: SD, standard deviation; BMI, body mass index; NHS3, National Hospital Seizure Severity Scale; AED, antiepileptic drug; NA, not applicable.

## References

[b1] Moshe S. L., Perucca E., Ryvlin P. & Tomson T. Epilepsy: new advances. Lancet doi:10.1016/S0140-6736(14)60456-6 (2014). 25260236

[b2] Devinsky O. Patients with Refractory Seizures. N. Engl. J. Med. 340, 1565–1570 (1993). 10.1056/NEJM199905203402008 10332020

[b3] Kwan P. & Brodie M. J. Early identification of refractory epilepsy. N. Engl. J. Med. 342, 314–319 (2000). 10660394 10.1056/NEJM200002033420503

[b4] Chen X. *et al.* Characterization of microRNAs in serum: a novel class of biomarkers for diagnosis of cancer and other diseases. Cell Res. 18, 997–1006 (2008). 18766170 10.1038/cr.2008.282

[b5] ElSharawy A. *et al.* Genome-wide miRNA signatures of human longevity. Aging. Cell 11, 607–616 (2012). 22533606 10.1111/j.1474-9726.2012.00824.x

[b6] Mitchell P. S. *et al.* Circulating microRNAs as stable blood-based markers for cancer detection. Proc. Natl. Acad. Sci. USA 105, 10513–10518 (2008). 18663219 10.1073/pnas.0804549105 PMC2492472

[b7] Cardo L. F. *et al.* Profile of microRNAs in the plasma of Parkinson’s disease patients and healthy controls. J. Neurol. 260, 1420–1422 (2013). 23543376 10.1007/s00415-013-6900-8

[b8] Gandhi R. *et al.* Circulating microRNAs as biomarkers for disease staging in multiple sclerosis. Ann. Neurol. 73, 729–740 (2013). 23494648 10.1002/ana.23880

[b9] Tan L. *et al.* Circulating miR-125b as a biomarker of Alzheimer’s disease. J. Neurol. Sci. 336, 52–56 (2013). 24139697 10.1016/j.jns.2013.10.002

[b10] Song Y. J. *et al.* Temporal lobe epilepsy induces differential expression of hippocampal miRNAs including let-7e and miR-23a/b. Brain Res. 1387, 134–140 (2011). 21376023 10.1016/j.brainres.2011.02.073

[b11] Hu K. *et al.* MicroRNA expression profile of the hippocampus in a rat model of temporal lobe epilepsy and miR-34a-targeted neuroprotection against hippocampal neurone cell apoptosis post-status epilepticus. BMC Neurosci. 13, 115 (2012). 22998082 10.1186/1471-2202-13-115 PMC3471047

[b12] Kan A. A. *et al.* Genome-wide microRNA profiling of human temporal lobe epilepsy identifies modulators of the immune response. Cell. Mol. Life Sci. 69, 3127–3145 (2012). 22535415 10.1007/s00018-012-0992-7 PMC3428527

[b13] McKiernan R. C. *et al.* Reduced mature microRNA levels in association with dicer loss in human temporal lobe epilepsy with hippocampal sclerosis. PLoS One 7, e35921 (2012). 22615744 10.1371/journal.pone.0035921 PMC3352899

[b14] Bot A. M., Debski K. J. & Lukasiuk K. Alterations in miRNA levels in the dentate gyrus in epileptic rats. PLoS One 8, e76051 (2013). 24146813 10.1371/journal.pone.0076051 PMC3795667

[b15] Gorter J. A. *et al.* Hippocampal subregion-specific microRNA expression during epileptogenesis in experimental temporal lobe epilepsy. Neurobiol. Dis. 62, 508–520 (2014). 24184920 10.1016/j.nbd.2013.10.026

[b16] Li M. M. *et al.* Genome-wide microRNA expression profiles in hippocampus of rats with chronic temporal lobe epilepsy. Sci. Rep. 4, 4734 (2014). 24751812 10.1038/srep04734 PMC3994440

[b17] Sun Z. *et al.* Genome-wide microRNA profiling of rat hippocampus after status epilepticus induced by amygdala stimulation identifies modulators of neuronal apoptosis. PLoS One 8, e78375 (2013). 24205215 10.1371/journal.pone.0078375 PMC3808371

[b18] Henshall D. C. MicroRNA and epilepsy: profiling, functions and potential clinical applications. Curr Opin Neurol 27, 199–205 (2014). 24553459 10.1097/WCO.0000000000000079 PMC4127484

[b19] Iyer A. *et al.* MicroRNA-146a: a key regulator of astrocyte-mediated inflammatory response. PLoS One 7, e44789 (2012). 23028621 10.1371/journal.pone.0044789 PMC3441440

[b20] Tan L. *et al.* Genome-wide serum microRNA expression profiling identifies serum biomarkers for Alzheimer’s disease. J. Alzheimers. Dis. 40, 1017–1027 (2014). 24577456 10.3233/JAD-132144

[b21] Proper E. A. *et al.* Distribution of glutamate transporters in the hippocampus of patients with pharmaco-resistant temporal lobe epilepsy. Brain 125, 32–43 (2002). 11834591 10.1093/brain/awf001

[b22] Cross J. H., Boyd S. G., Gordon I., Harper A. & Neville B. G. Ictal cerebral perfusion related to EEG in drug resistant focal epilepsy of childhood. J. Neurol. Neurosurg. Psychiatry. 62, 377–384 (1997). 9120452 10.1136/jnnp.62.4.377 PMC1074095

[b23] Holmes G. L. EEG abnormalities as a biomarker for cognitive comorbidities in pharmacoresistant epilepsy. Epilepsia 54 **Suppl 2**, 60–62 (2013). 23646973 10.1111/epi.12186 PMC4682563

[b24] Aronica E. *et al.* Expression pattern of miR-146a, an inflammation-associated microRNA, in experimental and human temporal lobe epilepsy. Eur. J. Neurosci. 31, 1100–1107 (2010). 20214679 10.1111/j.1460-9568.2010.07122.x

[b25] Nudelman A. S. *et al.* Neuronal activity rapidly induces transcription of the CREB-regulated microRNA-132, *in vivo*. Hippocampus 20, 492–498 (2010). 19557767 10.1002/hipo.20646 PMC2847008

[b26] Liu D. Z. *et al.* Brain and blood microRNA expression profiling of ischemic stroke, intracerebral hemorrhage, and kainate seizures. J. Cereb. Blood Flow Metab. 30, 92–101 (2010). 19724284 10.1038/jcbfm.2009.186 PMC2949089

[b27] Wieser H. G. & Epilepsy I. C. o. N. o. ILAE Commission Report. Mesial temporal lobe epilepsy with hippocampal sclerosis. Epilepsia 45, 695–714 (2004). 15144438 10.1111/j.0013-9580.2004.09004.x

[b28] Lu Z. *et al.* miR-301a as an NF-kappaB activator in pancreatic cancer cells. EMBO J. 30, 57–67 (2011). 21113131 10.1038/emboj.2010.296 PMC3020116

[b29] Kumar P. *et al.* Circulating miRNA biomarkers for Alzheimer’s disease. PLoS One 8, e69807 (2013). 23922807 10.1371/journal.pone.0069807 PMC3726785

[b30] Kosik K. S. The neuronal microRNA system. Nat. Rev. Neurosci. 7, 911–920 (2006). 17115073 10.1038/nrn2037

[b31] Wayman G. A. *et al.* An activity-regulated microRNA controls dendritic plasticity by down-regulating p250GAP. Proc. Natl. Acad. Sci. USA 105, 9093–9098 (2008). 18577589 10.1073/pnas.0803072105 PMC2449370

[b32] Edbauer D. *et al.* Regulation of synaptic structure and function by FMRP-associated microRNAs miR-125b and miR-132. Neuron 65, 373–384 (2010). 20159450 10.1016/j.neuron.2010.01.005 PMC5018398

[b33] Gaughwin P., Ciesla M., Yang H., Lim B. & Brundin P. Stage-specific modulation of cortical neuronal development by Mmu-miR-134. Cereb. Cortex. 21, 1857–1869 (2011). 21228099 10.1093/cercor/bhq262

[b34] Tao J. *et al.* Deletion of astroglial Dicer causes non-cell-autonomous neuronal dysfunction and degeneration. J. Neurosci. 31, 8306–8319 (2011). 21632951 10.1523/JNEUROSCI.0567-11.2011 PMC3500097

[b35] O’Donoghue M. F., Duncan J. S. & Sander J. W. The National Hospital Seizure Severity Scale: a further development of the Chalfont Seizure Severity Scale. Epilepsia 37, 563–571 (1996). 8641234 10.1111/j.1528-1157.1996.tb00610.x

[b36] Engel J. Jr. A proposed diagnostic scheme for people with epileptic seizures and with epilepsy: report of the ILAE Task Force on Classification and Terminology. Epilepsia 42, 796–803 (2001). 11422340 10.1046/j.1528-1157.2001.10401.x

[b37] Kwan P. *et al.* Definition of drug resistant epilepsy: consensus proposal by the ad hoc Task Force of the ILAE Commission on Therapeutic Strategies. Epilepsia 51, 1069–1077 (2010). 19889013 10.1111/j.1528-1167.2009.02397.x

[b38] Yu S. *et al.* Circulating microRNA profiles as potential biomarkers for diagnosis of papillary thyroid carcinoma. J Clin Endocrinol Metab 97, 2084–2092 (2012). 22472564 10.1210/jc.2011-3059

[b39] Yang C. *et al.* Identification of seven serum microRNAs from a genome-wide serum microRNA expression profile as potential noninvasive biomarkers for malignant astrocytomas. Int J Cancer 132, 116–127 (2013). 22674182 10.1002/ijc.27657

[b40] McDonald J. S., Milosevic D., Reddi H. V., Grebe S. K. & Algeciras-Schimnich A. Analysis of circulating microRNA: preanalytical and analytical challenges. Clin. Chem. 57, 833–840 (2011). 21487102 10.1373/clinchem.2010.157198

[b41] Livak K. J. & Schmittgen T. D. Analysis of relative gene expression data using real-time quantitative PCR and the 2(-Delta Delta C(T)) Method. Methods 25, 402–408 (2001). 11846609 10.1006/meth.2001.1262

