# Supplementary material for: Circulating microRNAs are promising novel biomarkers for drug-resistant epilepsy
Source: Sci Rep. 2015 May 18;5:10201. doi: 10.1038/srep10201 (PMC4435024; doi:10.1038/srep10201)
Supplement: Supporting Information [file srep10201-s1.pdf]

Circulating microRNAs are promising novel biomarkers for drug-resistant epilepsy

Jun Wang, Lan Tan, Lin Tan, Yan Tian, Jing Ma, Chen-Chen Tan, Hui-Fu Wang, Ying Liu,

Meng-Shan Tan, Teng Jiang, Jin-Tai Yu

List of contents:

Supplementary Table S1. Differentially exhpressed miRNAs in Bug-resistant epilepsy samples compared to Bug-responsive epilepsy samples by Illumina Hiseq2000 sequencing.

Supplementary Table S2. Mean Cq values of miRNAs in qRT-PCR in training phase.

Supplementary Table S3. The primer sequences of selected miRNAs in discovery phase for real-time PCR.

Supplementary Table S4. Mean Cq values of miRNAs in qRT-PCR in validation phase.

Supplementary Figure S1. A network of genes potentially targeted by five differentially expressed miRNAs.

Supplementary Table S1.Differentially expressed miRNAs in Bug-resistant epilepsy samples compared to Bug-responsive epilepsy samples by Illumina Hiseq2000 sequencing.

pairwise	miR-name	A-expressed	B-expressed	A-std	B-std	fold-change(log2 B/A)	p-value	sig-lable
A-B	hsa-miR-194-5p	100	18	10.4814	1.8736	-2.48394654	2.52E-15	**
A-B	hsa-miR-204-5p	40	6	4.1926	0.6245	-2.74707172	1.57E-07	**
A-B	hsa-miR-221-5p	13	3	1.3532	0.3115	-2.11907101	0.012547	*
A-B	hsa-miR-301a-3p	304	66	31.8635	6.853	-2.21709715	2.53E-38	**
A-B	hsa-miR-30b-5p	62	14	6.4985	1.4573	-2.15680885	1.19E-08	**
A-B	hsa-miR-342-5p	39	7	4.0878	0.7286	-2.48812571	9.54E-07	**
A-B	hsa-miR-3605-5p	36	9	3.7733	0.9345	-2.01356024	3.54E-05	**
A-B	hsa-miR-4446-3p	16	4	1.677	0.4153	-2.01365691	0.0067539	**
A-B	hsa-miR-574-5p	10	44	1.0409	4.5687	2.13395224	2.15E-06	**
A-B	hsa-miR-598-3p	43	6	4.507	0.623	-2.85486338	2.71E-08	**
A-B	hsa-miR-874-3p	30	6	3.1444	0.623	-2.33548069	3.66E-05	**
A-B	hsa-miR-889-3p	55	8	5.7648	0.8307	-2.7948711	4.43E-10	**
A-B	novel_mir_451	33	7	3.435	0.7268	-2.24067977	2.45E-05	**
A-B	novel_mir_67	14	75	1.4674	7.7875	2.40789807	2.38E-11	**
A-B	novel_mir_9	13	55	1.3626	5.7108	2.06733075	2.06E-07	**
A-B	hsa-let-7a-3p	10	11	1.0409	1.1422	0.13398382	0.8363993	
A-B	hsa-let-7a-5p	5199	10431	541.1696	1083.087	1.00099643	0	**
A-B	hsa-let-7b-5p	3638	4660	378.6834	483.864	0.35360942	1.00E-28	
A-B	hsa-let-7c-5p	392	797	40.8037	82.7553	1.02015173	3.25E-32	**
A-B	hsa-let-7d-3p	123	137	12.8032	14.2252	0.1519445	0.3972869	
A-B	hsa-let-7d-5p	105	181	10.9296	18.7939	0.78202388	7.09E-06	
A-B	hsa-let-7e-5p	53	70	5.5168	7.2683	0.39778629	0.1299734	
A-B	hsa-let-7f-5p	12300	21731	1280.321	2256.405	0.81752118	0	

A-B	hsa-let-7g-3p	24	8	2.4982	0.8307	-1.58848952	0.0044522	**
A-B	hsa-let-7g-5p	1427	1803	148.538	187.2118	0.33383931	5.68E-11	
A-B	hsa-let-7i-3p	28	16	2.9146	1.6613	-0.81098528	0.0711433	
A-B	hsa-let-7i-5p	12172	11173	1266.997	1160.132	-0.12712465	1.71E-11	
A-B	hsa-miR-100-5p	11197	10149	1165.508	1053.806	-0.14534973	1.91E-13	
A-B	hsa-miR-101-3p	4261	4035	443.5322	418.9681	-0.08219844	0.0094837	
A-B	hsa-miR-103a-3p	11703	13511	1218.178	1402.894	0.20368102	4.36E-29	
A-B	hsa-miR-106b-3p	3607	3031	375.4566	314.7193	-0.2545805	7.26E-13	
A-B	hsa-miR-106b-5p	83	59	8.6396	6.1262	-0.49597205	0.0428306	
A-B	hsa-miR-107	11277	12930	1173.835	1342.567	0.19376386	1.71E-25	
A-B	hsa-miR-10a-5p	106309	117521	11065.82	12202.61	0.14107977	3.09E-118	
A-B	hsa-miR-10b-5p	1086134	989551	113056.9	102748.5	-0.1379312	0	
A-B	hsa-miR-1180-3p	36	20	3.7473	2.0767	-0.85155866	0.0323816	
A-B	hsa-miR-122-3p	0	18	0.01	1.869	7.54612276	4.17E-06	**
A-B	hsa-miR-1224-5p	6	13	0.6245	1.3498	1.11197218	0.1166063	
A-B	hsa-miR-122-5p	1318	3534	137.1921	366.9475	1.41937626	3.23E-229	**
A-B	hsa-miR-1246	16	25	1.6655	2.5958	0.64022388	0.1666023	
A-B	hsa-miR-125a-3p	10	0	1.0409	0.01	-6.70168766	0.0009633	**
A-B	hsa-miR-125a-5p	654	975	68.0756	101.2376	0.57253551	2.25E-15	
A-B	hsa-miR-125b-2-3p	243	300	25.2941	31.15	0.30043126	0.015656	
A-B	hsa-miR-125b-5p	54	113	5.6209	11.7332	1.06172348	4.59E-06	**
A-B	hsa-miR-126-3p	231	200	24.045	20.7667	-0.21146495	0.129116	
A-B	hsa-miR-126-5p	1723	2449	179.349	254.2882	0.50369482	4.71E-29	
A-B	hsa-miR-1271-5p	10	25	1.0409	2.5958	1.31834776	0.0115715	*
A-B	hsa-miR-1273h-3p	23	19	2.3941	1.9728	-0.27923871	0.5369852	
A-B	hsa-miR-127-3p	333	241	34.6623	25.0239	-0.47006073	0.0001071	

A-B	hsa-miR-128-1-5p	25	12	2.6023	1.246	-1.06248322	0.0329229	*
A-B	hsa-miR-128-3p	42	108	4.3718	11.214	1.35900167	5.18E-08	**
A-B	hsa-miR-1285-3p	17	30	1.7695	3.115	0.8158904	0.0606311	
A-B	hsa-miR-1304-3p	0	21	0.01	2.1805	7.76851518	5.29E-07	**
A-B	hsa-miR-1307-3p	85	64	8.8477	6.6453	-0.41296814	0.0833319	
A-B	hsa-miR-1307-5p	176	169	18.32	17.5479	-0.06212111	0.6896669	
A-B	hsa-miR-130a-3p	694	866	72.2392	89.9198	0.31585691	1.64E-05	
A-B	hsa-miR-130b-3p	166	201	17.2791	20.8705	0.27243709	0.0715928	
A-B	hsa-miR-130b-5p	12	8	1.2491	0.8307	-0.58848952	0.3801922	
A-B	hsa-miR-134-5p	44	23	4.58	2.3882	-0.93942394	0.0100386	
A-B	hsa-miR-136-3p	6	15	0.6245	1.5575	1.31845869	0.053188	
A-B	hsa-miR-139-5p	68	117	7.0782	12.1485	0.77932376	0.0003235	
A-B	hsa-miR-140-3p	941	975	97.9497	101.2376	0.04763223	0.4701227	
A-B	hsa-miR-141-3p	82	91	8.5355	9.4488	0.14665545	0.5055727	
A-B	hsa-miR-142-3p	104	41	10.8255	4.2572	-1.34645689	1.08E-07	**
A-B	hsa-miR-142-5p	5683	8328	591.5497	864.7252	0.54774235	7.96E-110	
A-B	hsa-miR-143-3p	108020	100333	11243.92	10417.92	-0.11007731	7.18E-68	
A-B	hsa-miR-144-3p	121	220	12.595	22.8434	0.85892627	7.97E-08	
A-B	hsa-miR-144-5p	266	274	27.6882	28.4504	0.03917767	0.7527098	
A-B	hsa-miR-145-3p	51	55	5.3086	5.7108	0.10536142	0.7087098	
A-B	hsa-miR-145-5p	286	126	29.7701	13.083	-1.18617067	1.24E-15	**
A-B	hsa-miR-1468-5p	18	20	1.8736	2.0767	0.14847984	0.7551687	
A-B	hsa-miR-146a-5p	6770	5031	704.6968	522.3862	-0.43188588	8.09E-59	
A-B	hsa-miR-146b-3p	41	55	4.2677	5.7108	0.4202341	0.1583885	
A-B	hsa-miR-146b-5p	3544	5842	368.8989	606.5952	0.71750859	2.25E-124	
A-B	hsa-miR-148a-3p	6938	14607	722.1841	1516.696	1.070493	0	**

A-B	hsa-miR-148a-5p	183	340	19.0487	35.3034	0.89011459	5.89E-12	
A-B	hsa-miR-148b-3p	560	360	58.291	37.3801	-0.64100272	2.86E-11	
A-B	hsa-miR-148b-5p	10	4	1.0409	0.4153	-1.3256057	0.117338	
A-B	hsa-miR-150-3p	27	76	2.8105	7.8913	1.48943617	1.03E-06	**
A-B	hsa-miR-150-5p	257	274	26.7514	28.4504	0.08883454	0.4786961	
A-B	hsa-miR-151a-3p	8829	11257	919.0203	1168.854	0.34692548	1.22E-64	
A-B	hsa-miR-151a-5p	439	897	45.696	93.1386	1.02731131	1.91E-36	**
A-B	hsa-miR-151b	63	111	6.5577	11.5255	0.81356753	0.0002793	
A-B	hsa-miR-152-3p	22	9	2.29	0.9345	-1.29308103	0.0196924	*
A-B	hsa-miR-155-5p	0	45	0.01	4.6725	8.86805085	3.01E-14	**
A-B	hsa-miR-15a-5p	462	834	48.0901	86.5971	0.84857879	3.88E-25	
A-B	hsa-miR-15b-3p	108	50	11.2418	5.1917	-1.11459413	3.07E-06	**
A-B	hsa-miR-15b-5p	40	32	4.1636	3.3227	-0.32547543	0.3437461	
A-B	hsa-miR-16-2-3p	249	213	25.9187	22.1165	-0.22887027	0.0890809	
A-B	hsa-miR-16-5p	5748	10953	598.3157	1137.288	0.92661894	0	
A-B	hsa-miR-17-3p	29	37	3.0186	3.8418	0.34790282	0.3334894	
A-B	hsa-miR-17-5p	49	55	5.1005	5.7108	0.16305418	0.5669797	
A-B	hsa-miR-181a-2-3p	418	466	43.5101	48.3864	0.15325127	0.1148219	
A-B	hsa-miR-181a-3p	151	115	15.7178	11.9409	-0.39648772	0.0260099	
A-B	hsa-miR-181a-5p	15911	22186	1656.194	2303.65	0.47605005	2.02E-224	
A-B	hsa-miR-181b-5p	289	555	30.0823	57.6276	0.93784506	3.84E-20	
A-B	hsa-miR-181c-3p	43	46	4.4759	4.7763	0.09371565	0.7609956	
A-B	hsa-miR-181c-5p	260	170	27.0637	17.6517	-0.61655196	1.21E-05	
A-B	hsa-miR-181d-5p	280	596	29.348	61.8848	1.07632291	1.69E-26	**
A-B	hsa-miR-182-5p	29904	38390	3112.74	3986.167	0.35681705	2.39E-227	
A-B	hsa-miR-183-5p	2770	3653	288.3324	379.3037	0.39562048	7.84E-28	

A-B	hsa-miR-184	17	8	1.7695	0.8307	-1.0909423	0.0744861	
A-B	hsa-miR-185-3p	5	11	0.5205	1.1422	1.13384522	0.1448722	
A-B	hsa-miR-185-5p	156	84	16.2382	8.722	-0.89666082	2.72E-06	
A-B	hsa-miR-186-5p	3866	4491	402.4162	466.3162	0.21262017	1.74E-11	
A-B	hsa-miR-190b	12	5	1.2491	0.5192	-1.26652669	0.0952041	
A-B	hsa-miR-191-5p	19294	21890	2008.334	2272.915	0.17854467	4.25E-36	
A-B	hsa-miR-192-5p	37561	71404	3909.766	7414.126	0.92319437	0	
A-B	hsa-miR-193a-5p	42	17	4.4022	1.7652	-1.31839304	0.0009377	**
A-B	hsa-miR-193b-3p	27	30	2.8105	3.115	0.14840535	0.7010138	
A-B	hsa-miR-193b-5p	16	6	1.6655	0.623	-1.41865129	0.0341859	*
A-B	hsa-miR-194-3p	0	11	0.01	1.1422	6.83567148	0.0004956	**
A-B	hsa-miR-195-3p	0	11	0.01	1.1422	6.83567148	0.0004956	**
A-B	hsa-miR-197-3p	143	240	14.885	24.92	0.74344485	7.36E-07	
A-B	hsa-miR-199a-3p	382	347	39.7628	36.0302	-0.14221269	0.1838513	
A-B	hsa-miR-199a-5p	67	69	6.9741	7.1645	0.03885897	0.8758431	
A-B	hsa-miR-199b-3p	382	347	39.7628	36.0302	-0.14221269	0.1838513	
A-B	hsa-miR-199b-5p	7	12	0.7286	1.246	0.77410517	0.2655649	
A-B	hsa-miR-19a-3p	831	426	86.4997	44.2331	-0.96756877	6.28E-31	
A-B	hsa-miR-19b-3p	2949	1724	306.9647	179.0089	-0.77804144	2.80E-73	
A-B	hsa-miR-203a	0	14	0.01	1.4537	7.18358576	6.22E-05	**
A-B	hsa-miR-20a-5p	25	22	2.6023	2.2843	-0.18803516	0.659209	
A-B	hsa-miR-210-3p	109	85	11.3459	8.8258	-0.36237209	0.0823136	
A-B	hsa-miR-2110	20	13	2.0818	1.3498	-0.62508582	0.2266823	
A-B	hsa-miR-21-3p	1101	1081	114.6043	112.244	-0.03002285	0.6270085	
A-B	hsa-miR-215-5p	791	891	82.3361	92.5156	0.16817154	0.0169703	
A-B	hsa-miR-21-5p	3810	5522	396.5871	573.3685	0.5318249	9.82E-70	

A-B	hsa-miR-221-3p	1313	1021	136.6716	106.014	-0.36645869	9.94E-10	
A-B	hsa-miR-222-3p	154	295	16.03	30.6309	0.93421333	2.46E-11	
A-B	hsa-miR-223-3p	360	725	37.4728	75.2793	1.00640943	7.72E-29	**
A-B	hsa-miR-223-5p	69	102	7.1823	10.591	0.56032099	0.0121903	
A-B	hsa-miR-22-3p	20128	23260	2095.146	2415.167	0.2050722	1.84E-49	
A-B	hsa-miR-224-5p	52	41	5.4503	4.2572	-0.35643077	0.2378076	
A-B	hsa-miR-23a-3p	412	697	42.8855	72.3719	0.75493971	1.12E-17	
A-B	hsa-miR-23b-3p	85	218	8.8477	22.6357	1.35522555	1.03E-14	**
A-B	hsa-miR-24-3p	946	525	98.4702	54.5126	-0.85309746	1.30E-28	
A-B	hsa-miR-25-3p	28033	40836	2917.986	4240.144	0.53914035	0	
A-B	hsa-miR-25-5p	0	11	0.01	1.1422	6.83567148	0.0004956	**
A-B	hsa-miR-26a-5p	2669	4741	277.8192	492.2745	0.8253167	1.41E-128	
A-B	hsa-miR-26b-3p	47	75	4.8923	7.7875	0.67064738	0.0117247	
A-B	hsa-miR-26b-5p	389	588	40.4914	61.0541	0.59247266	2.20E-10	
A-B	hsa-miR-27a-3p	534	608	55.5846	63.1308	0.1836588	0.0317507	
A-B	hsa-miR-27a-5p	7	10	0.7286	1.0383	0.51102445	0.4839977	
A-B	hsa-miR-27b-3p	5069	8633	527.6378	896.3944	0.76458571	5.18E-204	
A-B	hsa-miR-27b-5p	7	21	0.7286	2.1805	1.58146009	0.0082899	**
A-B	hsa-miR-28-3p	2351	4196	244.7182	435.6853	0.83216504	1.28E-115	
A-B	hsa-miR-28-5p	22	26	2.29	2.6997	0.2374515	0.5740939	
A-B	hsa-miR-29a-3p	102	116	10.6173	12.0447	0.18198153	0.3535934	
A-B	hsa-miR-30a-3p	216	468	22.4837	48.5941	1.11190169	2.75E-22	**
A-B	hsa-miR-30a-5p	4081	3884	424.7958	403.2892	-0.07495473	0.020455	
A-B	hsa-miR-30c-1-3p	46	14	4.8215	1.4537	-1.72975248	2.32E-05	**
A-B	hsa-miR-30c-2-3p	23	41	2.3941	4.2572	0.83042145	0.0254597	
A-B	hsa-miR-30c-5p	73	79	7.5987	8.2028	0.11036383	0.6386996	

A-B	hsa-miR-30d-3p	25	18	2.6023	1.869	-0.47752072	0.2874611	
A-B	hsa-miR-30d-5p	2577	4169	268.2428	432.8818	0.6904337	1.34E-83	
A-B	hsa-miR-30e-3p	769	1786	80.0461	185.4466	1.2121008	6.27E-92	**
A-B	hsa-miR-30e-5p	1344	1366	139.8984	141.8365	0.01984938	0.720342	
A-B	hsa-miR-3131	11	0	1.145	0.01	-6.83920378	0.0004811	**
A-B	hsa-miR-3150b-3p	19	24	1.9777	2.492	0.33348047	0.4563574	
A-B	hsa-miR-3158-3p	10	25	1.0409	2.5958	1.31834776	0.0115715	*
A-B	hsa-miR-3200-3p	20	9	2.0818	0.9345	-1.15556491	0.0420853	*
A-B	hsa-miR-320a	3581	1873	372.7502	194.4801	-0.93858659	3.24E-121	
A-B	hsa-miR-320b	711	438	74.0088	45.4791	-0.70249312	4.29E-16	
A-B	hsa-miR-320c	41	61	4.2677	6.3338	0.56961255	0.0496765	
A-B	hsa-miR-320d	0	13	0.01	1.3498	7.07660185	0.0001304	**
A-B	hsa-miR-323b-3p	12	6	1.2491	0.623	-1.00358491	0.1654076	
A-B	hsa-miR-324-3p	0	12	0.01	1.246	6.96116026	0.0002595	**
A-B	hsa-miR-324-5p	13	0	1.3532	0.01	-7.08023128	0.00012	**
A-B	hsa-miR-32-5p	12	18	1.2491	1.869	0.58137759	0.284147	
A-B	hsa-miR-331-3p	8	28	0.8327	2.9073	1.80381122	0.0007733	**
A-B	hsa-miR-335-3p	5	10	0.5205	1.0383	0.99625328	0.2119359	
A-B	hsa-miR-335-5p	38	27	3.9555	2.8035	-0.496631	0.1720977	
A-B	hsa-miR-338-5p	0	16	0.01	1.6613	7.37616881	1.56E-05	**
A-B	hsa-miR-339-3p	22	17	2.29	1.7652	-0.37551595	0.4250052	
A-B	hsa-miR-339-5p	104	56	10.8255	5.8147	-0.896657	0.0001305	
A-B	hsa-miR-33b-5p	14	12	1.4573	1.246	-0.22599383	0.6962997	
A-B	hsa-miR-340-5p	354	463	36.8483	48.0749	0.38368579	0.0001562	
A-B	hsa-miR-342-3p	593	596	61.726	61.8848	0.0037068	0.9647379	
A-B	hsa-miR-345-5p	162	140	16.8628	14.5367	-0.21414431	0.1985654	

A-B	hsa-miR-3605-3p	0	19	0.01	1.9728	7.62410089	2.09E-06	**
A-B	hsa-miR-361-3p	36	29	3.7473	3.0112	-0.31551295	0.3835185	
A-B	hsa-miR-3614-5p	10	0	1.0481	0.01	-6.71163257	0.0009272	**
A-B	hsa-miR-3615	64	65	6.6618	6.7492	0.01880446	0.941412	
A-B	hsa-miR-361-5p	32	62	3.3309	6.4377	0.95063331	0.0020038	
A-B	hsa-miR-363-3p	635	698	66.0979	72.4758	0.13289492	0.0929857	
A-B	hsa-miR-365a-3p	4	10	0.4164	1.0383	1.31818137	0.119609	
A-B	hsa-miR-365b-3p	4	10	0.4164	1.0383	1.31818137	0.119609	
A-B	hsa-miR-3675-5p	0	13	0.01	1.3498	7.07660185	0.0001304	**
A-B	hsa-miR-3688-3p	29	18	3.0186	1.869	-0.69161303	0.1094854	
A-B	hsa-miR-370-3p	17	0	1.7695	0.01	-7.46719796	7.46E-06	**
A-B	hsa-miR-371b-5p	21	22	2.1859	2.2843	0.06352473	0.8869202	
A-B	hsa-miR-374a-3p	6	13	0.6245	1.3498	1.11197218	0.1166063	
A-B	hsa-miR-374a-5p	0	12	0.01	1.246	6.96116026	0.0002481	**
A-B	hsa-miR-374b-5p	19	0	1.9915	0.01	-7.63771167	1.74E-06	**
A-B	hsa-miR-375	1559	2255	162.278	234.1445	0.52893172	3.13E-29	
A-B	hsa-miR-377-5p	12	0	1.2491	0.01	-6.96474516	0.0002402	**
A-B	hsa-miR-378a-3p	3360	2714	349.7461	281.8041	-0.31161537	4.76E-17	
A-B	hsa-miR-378c	163	66	16.9668	6.853	-1.3079069	7.34E-11	**
A-B	hsa-miR-378d	10	0	1.0409	0.01	-6.70168766	0.0009633	**
A-B	hsa-miR-381-3p	53	39	5.5168	4.0495	-0.44608789	0.1428863	
A-B	hsa-miR-3912-3p	0	13	0.01	1.3498	7.07660185	0.0001242	**
A-B	hsa-miR-409-3p	322	648	33.5173	67.2841	1.00535968	6.62E-26	**
A-B	hsa-miR-409-5p	10	8	1.0409	0.8307	-0.32543202	0.6436812	
A-B	hsa-miR-410-3p	81	95	8.4314	9.8642	0.22642985	0.3002696	
A-B	hsa-miR-411-5p	20	31	2.0818	3.2188	0.62869146	0.1285807	

A-B	hsa-miR-412-5p	24	0	2.4982	0.01	-7.96474516	5.78E-08	**
A-B	hsa-miR-421	42	108	4.3718	11.214	1.35900167	5.18E-08	**
A-B	hsa-miR-423-3p	1492	1845	155.3039	191.5728	0.30279868	1.50E-09	
A-B	hsa-miR-423-5p	11826	8740	1230.981	907.5046	-0.4398321	9.29E-105	
A-B	hsa-miR-424-3p	29	78	3.0186	8.099	1.42386419	1.70E-06	**
A-B	hsa-miR-424-5p	104	261	10.9007	27.1005	1.31389869	1.57E-16	**
A-B	hsa-miR-425-3p	25	27	2.6023	2.8035	0.10744178	0.7908181	
A-B	hsa-miR-425-5p	401	442	41.7405	45.8944	0.13687025	0.1690021	
A-B	hsa-miR-4286	9	19	0.9368	1.9728	1.07443172	0.0623586	
A-B	hsa-miR-431-5p	9	12	0.9368	1.246	0.41149109	0.5272946	
A-B	hsa-miR-432-5p	54	53	5.6209	5.5032	-0.03053039	0.9131576	
A-B	hsa-miR-4433b-5p	4	15	0.4164	1.5575	1.90319019	0.0120024	*
A-B	hsa-miR-4504	12	9	1.2491	0.9345	-0.41862241	0.5196537	
A-B	hsa-miR-450b-5p	12	27	1.2491	2.8035	1.16634009	0.0169449	*
A-B	hsa-miR-4511	0	13	0.01	1.3498	7.07660185	0.0001304	**
A-B	hsa-miR-451a	19605	20878	2040.706	2167.835	0.0871868	1.22E-09	
A-B	hsa-miR-454-3p	0	14	0.01	1.4537	7.18358576	6.22E-05	**
A-B	hsa-miR-454-5p	15	28	1.5614	2.9073	0.89683977	0.0497135	
A-B	hsa-miR-4645-3p	0	12	0.01	1.246	6.96116026	0.0002481	**
A-B	hsa-miR-4677-3p	85	64	8.8477	6.6453	-0.41296814	0.0833319	
A-B	hsa-miR-4732-3p	33	38	3.435	3.9457	0.19997117	0.5630947	
A-B	hsa-miR-4732-5p	26	20	2.7064	2.0767	-0.38208226	0.3770695	
A-B	hsa-miR-483-3p	9	13	0.9368	1.3498	0.52693268	0.4082609	
A-B	hsa-miR-484	102	222	10.6173	23.051	1.11841241	1.92E-11	**
A-B	hsa-miR-485-5p	25	7	2.6204	0.7268	-1.85015673	0.0012022	**
A-B	hsa-miR-486-3p	250	447	26.0228	46.4136	0.8347714	7.77E-14	

A-B	hsa-miR-486-5p	3667940	3750915	381799.9	389470.6	0.02869747	1.33E-161	
A-B	hsa-miR-487b-3p	7	10	0.7286	1.0383	0.51102445	0.4839977	
A-B	hsa-miR-493-3p	14	0	1.4573	0.01	-7.18715409	5.99E-05	**
A-B	hsa-miR-493-5p	12	39	1.2578	4.0495	1.68684125	0.0001459	**
A-B	hsa-miR-494-3p	0	14	0.01	1.4537	7.18358576	6.22E-05	**
A-B	hsa-miR-500a-3p	365	355	37.9933	36.8609	-0.04365373	0.6849903	
A-B	hsa-miR-5010-5p	0	16	0.01	1.6613	7.37616881	1.56E-05	**
A-B	hsa-miR-501-3p	2679	1867	278.8601	193.8571	-0.52454795	5.24E-34	
A-B	hsa-miR-502-3p	34	14	3.5391	1.4537	-1.28365296	0.0036989	**
A-B	hsa-miR-505-3p	13	24	1.3532	2.492	0.88092899	0.0741801	
A-B	hsa-miR-5189-5p	14	5	1.4674	0.5192	-1.4988999	0.0393481	*
A-B	hsa-miR-532-3p	14	7	1.4573	0.7268	-1.00366758	0.1322957	
A-B	hsa-miR-532-5p	1919	1668	199.7508	173.1942	-0.20581066	1.99E-05	
A-B	hsa-miR-542-3p	0	16	0.01	1.6613	7.37616881	1.56E-05	**
A-B	hsa-miR-548am-5p	4	12	0.4164	1.246	1.58126209	0.0496296	*
A-B	hsa-miR-548au-5p	4	12	0.4164	1.246	1.58126209	0.0496296	*
A-B	hsa-miR-548c-5p	4	12	0.4164	1.246	1.58126209	0.0496296	*
A-B	hsa-miR-548k	16	17	1.6655	1.7652	0.0838763	0.869892	
A-B	hsa-miR-548o-3p	6	17	0.6245	1.7652	1.49905818	0.0230161	*
A-B	hsa-miR-548o-5p	4	12	0.4164	1.246	1.58126209	0.0496296	*
A-B	hsa-miR-5683	18	0	1.8736	0.01	-7.54966918	3.73E-06	**
A-B	hsa-miR-574-3p	54	34	5.6209	3.5303	-0.67101036	0.0324589	
A-B	hsa-miR-576-5p	0	13	0.01	1.3498	7.07660185	0.0001242	**
A-B	hsa-miR-582-3p	87	162	9.0559	16.821	0.89333354	1.94E-06	
A-B	hsa-miR-584-5p	123	261	12.8032	27.1005	1.08181503	1.38E-12	**
A-B	hsa-miR-589-5p	131	97	13.6359	10.0718	-0.43708838	0.0232773	

A-B	hsa-miR-625-3p	36	44	3.7473	4.5687	0.28593223	0.3802125	
A-B	hsa-miR-629-5p	41	32	4.2677	3.3227	-0.36110272	0.2905109	
A-B	hsa-miR-6503-3p	13	6	1.3532	0.623	-1.11907101	0.1140415	
A-B	hsa-miR-6514-5p	0	14	0.01	1.4537	7.18358576	6.22E-05	**
A-B	hsa-miR-652-3p	125	83	13.0114	8.6182	-0.59431772	0.0033698	
A-B	hsa-miR-654-3p	12	30	1.2491	3.115	1.31834318	0.005537	**
A-B	hsa-miR-654-5p	11	0	1.145	0.01	-6.83920378	0.0004811	**
A-B	hsa-miR-660-5p	132	150	13.74	15.575	0.18085016	0.294096	
A-B	hsa-miR-664a-5p	9	19	0.9368	1.9728	1.07443172	0.0623586	
A-B	hsa-miR-671-3p	5	12	0.5205	1.246	1.259334	0.0973099	
A-B	hsa-miR-675-3p	10	0	1.0409	0.01	-6.70168766	0.0009633	**
A-B	hsa-miR-6779-5p	10	0	1.0409	0.01	-6.70168766	0.0009633	**
A-B	hsa-miR-6837-3p	17	13	1.7695	1.3498	-0.3905961	0.4688563	
A-B	hsa-miR-6842-3p	18	6	1.8867	0.623	-1.59856097	0.0137167	*
A-B	hsa-miR-6852-5p	31	62	3.2268	6.4377	0.99644119	0.001315	
A-B	hsa-miR-744-5p	112	157	11.6582	16.3019	0.48369506	0.006453	
A-B	hsa-miR-7-5p	12	0	1.2491	0.01	-6.96474516	0.0002402	**
A-B	hsa-miR-7641	14	18	1.4573	1.869	0.35896867	0.4913494	
A-B	hsa-miR-766-3p	14	0	1.4573	0.01	-7.18715409	5.99E-05	**
A-B	hsa-miR-769-5p	551	368	57.3542	38.2107	-0.58592245	1.13E-09	
A-B	hsa-miR-7706	94	120	9.7846	12.46	0.34871929	0.0790036	
A-B	hsa-miR-7849-3p	12	11	1.2491	1.1422	-0.12907369	0.8340298	
A-B	hsa-miR-8061	0	13	0.01	1.3498	7.07660185	0.0001242	**
A-B	hsa-miR-873-3p	62	52	6.4536	5.3993	-0.25733178	0.3442933	
A-B	hsa-miR-873-5p	15	29	1.5614	3.0112	0.94749836	0.0364328	
A-B	hsa-miR-877-5p	14	15	1.4573	1.5575	0.09593426	0.8609087	

A-B	hsa-miR-885-5p	20	29	2.0818	3.0112	0.53250706	0.205768	
A-B	hsa-miR-92a-3p	7774	13184	809.2042	1368.941	0.75848413	3.47E-306	
A-B	hsa-miR-92b-3p	112	237	11.6582	24.6085	1.07781166	1.68E-11	**
A-B	hsa-miR-93-5p	1015	1360	105.6525	141.2135	0.41855111	2.07E-12	
A-B	hsa-miR-941	318	364	33.101	37.7954	0.19133586	0.0839503	
A-B	hsa-miR-9-5p	65	0	6.7659	0.01	-9.40213802	2.50E-20	**
A-B	hsa-miR-96-5p	132	78	13.74	8.099	-0.76256631	0.0001735	
A-B	hsa-miR-98-5p	465	616	48.4024	63.9614	0.40212293	5.19E-06	
A-B	hsa-miR-99a-5p	1187	1336	123.5561	138.7215	0.16702516	0.0036804	
A-B	hsa-miR-99b-5p	24755	15080	2576.775	1565.809	-0.71865859	0	
A-B	novel_mir_12	0	12	0.01	1.246	6.96116026	0.0002481	**
A-B	novel_mir_125	0	10	0.01	1.0383	6.69807954	0.00099	**
A-B	novel_mir_147	0	10	0.01	1.0383	6.69807954	0.00099	**
A-B	novel_mir_161	0	11	0.01	1.1422	6.83567148	0.0004956	**
A-B	novel_mir_164	27	0	2.8105	0.01	-8.13468302	7.20E-09	**
A-B	novel_mir_192	25	0	2.6023	0.01	-8.02364347	2.89E-08	**
A-B	novel_mir_247	21	0	2.1859	0.01	-7.77208359	4.64E-07	**
A-B	novel_mir_250	20	0	2.0818	0.01	-7.70168766	9.29E-07	**
A-B	novel_mir_253	12	0	1.2491	0.01	-6.96474516	0.0002402	**
A-B	novel_mir_261	14	14	1.4573	1.4537	-0.00356833	0.9946302	
A-B	novel_mir_279	34	0	3.5391	0.01	-8.46723873	5.57E-11	**
A-B	novel_mir_293	58	0	6.0373	0.01	-9.23775965	3.22E-18	**
A-B	novel_mir_295	0	15	0.01	1.5575	7.28308835	3.11E-05	**
A-B	novel_mir_30	110	65	11.45	6.7492	-0.76255919	0.0006133	
A-B	novel_mir_308	12	0	1.2491	0.01	-6.96474516	0.0002402	**
A-B	novel_mir_325	208	135	21.6509	14.0175	-0.62719793	7.10E-05	

A-B	novel_mir_332	19	12	1.9777	1.246	-0.66651953	0.2127329	
A-B	novel_mir_374	12	0	1.2491	0.01	-6.96474516	0.0002402	**
A-B	novel_mir_376	10	0	1.0409	0.01	-6.70168766	0.0009633	**
A-B	novel_mir_386	11	13	1.145	1.3498	0.23739806	0.6946522	
A-B	novel_mir_391	12	0	1.2491	0.01	-6.96474516	0.0002402	**
A-B	novel_mir_4	0	14	0.01	1.4537	7.18358576	6.22E-05	**
A-B	novel_mir_400	10	0	1.0409	0.01	-6.70168766	0.0009633	**
A-B	novel_mir_403	10	0	1.0409	0.01	-6.70168766	0.0009633	**
A-B	novel_mir_409	12	0	1.2491	0.01	-6.96474516	0.0002402	**
A-B	novel_mir_410	10	0	1.0409	0.01	-6.70168766	0.0009633	**
A-B	novel_mir_411	13	0	1.3532	0.01	-7.08023128	0.00012	**
A-B	novel_mir_414	15	18	1.5614	1.869	0.25942639	0.6126872	
A-B	novel_mir_418	14	0	1.4573	0.01	-7.18715409	5.99E-05	**
A-B	novel_mir_42	11	23	1.145	2.3882	1.06057606	0.0416872	*
A-B	novel_mir_423	29	11	3.0186	1.1422	-1.40206431	0.0042184	**
A-B	novel_mir_429	13	0	1.3532	0.01	-7.08023128	0.00012	**
A-B	novel_mir_431	11	0	1.145	0.01	-6.83920378	0.0004811	**
A-B	novel_mir_432	14	0	1.4573	0.01	-7.18715409	5.99E-05	**
A-B	novel_mir_435	11	0	1.145	0.01	-6.83920378	0.0004811	**
A-B	novel_mir_440	11	6	1.145	0.623	-0.87804353	0.2357852	
A-B	novel_mir_442	13	0	1.3532	0.01	-7.08023128	0.00012	**
A-B	novel_mir_443	10	5	1.0409	0.5192	-1.00346919	0.2083024	
A-B	novel_mir_449	13	0	1.3532	0.01	-7.08023128	0.00012	**
A-B	novel_mir_450	10	0	1.0409	0.01	-6.70168766	0.0009633	**
A-B	novel_mir_452	169	128	17.5914	13.2907	-0.40445321	0.0164156	
A-B	novel_mir_454	11	0	1.145	0.01	-6.83920378	0.0004811	**

A-B	novel_mir_457	12	0	1.2491	0.01	-6.96474516	0.0002402	**
A-B	novel_mir_459	14	0	1.4573	0.01	-7.18715409	5.99E-05	**
A-B	novel_mir_460	43	53	4.4759	5.5032	0.29809296	0.3157943	
A-B	novel_mir_461	10	0	1.0409	0.01	-6.70168766	0.0009633	**
A-B	novel_mir_462	11	0	1.145	0.01	-6.83920378	0.0004811	**
A-B	novel_mir_464	10	0	1.0409	0.01	-6.70168766	0.0009633	**
A-B	novel_mir_47	0	11	0.01	1.1422	6.83567148	0.0004956	**
A-B	novel_mir_470	12	0	1.2491	0.01	-6.96474516	0.0002402	**
A-B	novel_mir_471	11	0	1.145	0.01	-6.83920378	0.0004811	**
A-B	novel_mir_478	10	0	1.0409	0.01	-6.70168766	0.0009633	**
A-B	novel_mir_480	10	0	1.0409	0.01	-6.70168766	0.0009633	**
A-B	novel_mir_484	14	0	1.4573	0.01	-7.18715409	5.99E-05	**
A-B	novel_mir_487	12	0	1.2491	0.01	-6.96474516	0.0002402	**
A-B	novel_mir_488	13	0	1.3532	0.01	-7.08023128	0.00012	**
A-B	novel_mir_489	20	0	2.0818	0.01	-7.70168766	9.29E-07	**
A-B	novel_mir_490	10	0	1.0409	0.01	-6.70168766	0.0009633	**
A-B	novel_mir_495	13	0	1.3532	0.01	-7.08023128	0.00012	**
A-B	novel_mir_497	16	0	1.6655	0.01	-7.37981155	1.49E-05	**
A-B	novel_mir_498	12	0	1.2491	0.01	-6.96474516	0.0002402	**
A-B	novel_mir_501	10	0	1.0409	0.01	-6.70168766	0.0009633	**
A-B	novel_mir_504	14	0	1.4573	0.01	-7.18715409	5.99E-05	**
A-B	novel_mir_505	33	0	3.435	0.01	-8.42416626	1.12E-10	**
A-B	novel_mir_506	12	0	1.2491	0.01	-6.96474516	0.0002402	**
A-B	novel_mir_51	0	13	0.01	1.3498	7.07660185	0.0001242	**
A-B	novel_mir_510	10	0	1.0409	0.01	-6.70168766	0.0009633	**
A-B	novel_mir_518	10	0	1.0409	0.01	-6.70168766	0.0009633	**

A-B	novel_mir_519	13	0	1.3532	0.01	-7.08023128	0.00012	**
A-B	novel_mir_521	11	0	1.145	0.01	-6.83920378	0.0004811	**
A-B	novel_mir_530	50	20	5.2046	2.0767	-1.32549447	0.0002913	**
A-B	novel_mir_533	18	0	1.8736	0.01	-7.54966918	3.73E-06	**
A-B	novel_mir_543	0	11	0.01	1.1422	6.83567148	0.0004956	**
A-B	novel_mir_546	0	12	0.01	1.246	6.96116026	0.0002481	**
A-B	novel_mir_549	0	11	0.01	1.1422	6.83567148	0.0004956	**
A-B	novel_mir_557	0	11	0.01	1.1422	6.83567148	0.0004956	**
A-B	novel_mir_558	0	12	0.01	1.246	6.96116026	0.0002481	**
A-B	novel_mir_56	0	13	0.01	1.3498	7.07660185	0.0001242	**
A-B	novel_mir_580	0	85	0.01	8.8258	9.78558324	2.88E-26	**
A-B	novel_mir_586	0	10	0.01	1.0383	6.69807954	0.00099	**
A-B	novel_mir_589	0	10	0.01	1.0383	6.69807954	0.00099	**
A-B	novel_mir_596	0	14	0.01	1.4537	7.18358576	6.22E-05	**
A-B	novel_mir_600	0	11	0.01	1.1422	6.83567148	0.0004956	**
A-B	novel_mir_602	0	12	0.01	1.246	6.96116026	0.0002481	**
A-B	novel_mir_606	0	27	0.01	2.8035	8.13108526	7.71E-09	**
A-B	novel_mir_615	0	15	0.01	1.5575	7.28308835	3.11E-05	**
A-B	novel_mir_624	0	10	0.01	1.0383	6.69807954	0.00099	**
A-B	novel_mir_643	0	12	0.01	1.246	6.96116026	0.0002481	**
A-B	novel_mir_660	0	13	0.01	1.3498	7.07660185	0.0001242	**
A-B	novel_mir_661	0	10	0.01	1.0383	6.69807954	0.00099	**
A-B	novel_mir_73	0	14	0.01	1.4537	7.18358576	6.22E-05	**
A-B	novel_mir_96	14	16	1.4573	1.6613	0.18901472	0.7253086	
A-B	novel_mir_98	17	0	1.7695	0.01	-7.46719796	7.46E-06	**

Abbreviation: A represents drug-responsive epilepsy group; B represents drug-resistant epilepsy group.

Supplementary Table S2. Mean Cq values of miRNAs in qRT-PCR in the training phase.

sample	cel-miR-39	miR-194-5p	miR-301a-3p	miR-30b-5p	miR-342-5p	miR-4446-3p	novel-mir-67
A1	25.71	31.14	29.25	31.35	32.43	30.83	31.16
A2	25.82	29.42	28.66	32.00	31.02	30.85	32.60
A3	25.85	29.89	28.94	31.39	30.13	32.73	32.29
A4	25.76	30.00	28.98	30.46	29.69	30.59	31.86
A5	25.78	29.56	27.88	33.12	29.73	29.15	33.42
A6	25.34	31.65	27.53	29.62	33.51	32.37	32.68
A7	25.89	32.07	29.13	33.09	29.70	31.90	33.00
A8	25.87	31.70	29.87	32.17	30.18	31.46	33.67
A9	25.54	31.78	30.05	28.92	30.37	29.81	33.68
A10	24.15	28.58	28.32	27.39	29.20	29.93	31.41
A11	24.22	28.41	26.51	28.39	28.09	30.28	30.80
A12	24.41	28.70	27.22	28.42	28.44	29.80	32.64
A13	25.78	29.50	30.40		29.31	30.89	33.54
A14	25.87	34.56	28.56	30.52	31.92	33.48	33.12
A15	25.37	31.77	30.64	30.88	30.64	32.32	32.60
A16	25.10	30.08		31.01	29.58	30.54	32.94
A17	24.97	29.19	27.56	29.93	29.50	32.21	32.80
A18	24.11	27.95	26.57	28.43	29.54	28.24	32.28
A19	24.57	29.81	27.62	27.82	28.69	29.21	31.72
A20	24.05	28.47	26.48	27.31	29.68	31.22	30.22
A21	24.29	29.12	28.32	28.35	28.43	30.01	30.18
A22	25.51	32.28	28.09	30.89	30.70	30.46	
A23	25.69	32.99	28.95	32.16	31.18	31.17	33.30
A24	25.74	33.92	28.76	29.99	29.64	30.34	32.12
A25	25.25	28.90	28.62	30.72	30.04	31.03	31.41
A26	24.68	27.98	26.98	29.37	29.14	30.68	31.94
A27	24.58	27.90	26.90	28.67	28.93	29.67	32.75
A28	25.84	30.69	29.29	29.41	29.88	30.53	32.62
A29	25.85	31.28	29.79	32.87	29.70	32.78	31.98
A30	25.61	29.45	29.17	29.76	29.62	31.11	33.22
B1	25.68	31.74	31.99	33.94	31.92	29.17	33.24
B2	24.72	28.80	29.88	30.35	28.10	29.75	31.30
B3	25.07	29.08	29.35	31.66	31.65	32.08	30.25
B4	24.14	30.40	29.78	30.55	29.90	31.12	31.26
B5	25.07	30.77	31.40	34.66	34.41	32.91	29.83
B6	25.06	30.10	31.89	33.32	31.16	32.32	29.51
B7	24.95	29.73	30.95	30.10	31.01	29.76	28.79
B8	24.74	29.26	31.59	31.11	30.23	29.84	30.62
B9	25.24	29.64	30.50	30.37	30.73	34.37	29.66
B10	24.06	30.65	31.57	27.46	32.35		29.62
B11	25.47	31.23	30.35	31.48	31.82	32.16	32.47

B12	24.26	29.86	30.36	29.70	29.98	30.81	30.40
B13	24.59	32.56	29.89	32.56	32.55	31.95	29.99
B14	24.29	31.33	31.20	31.53	29.23	31.72	28.94
B15	24.15	31.26	30.01	31.35	31.39	31.40	30.71
B16	25.79	31.81	31.52	29.35	30.11	33.80	32.54
B17	25.58	34.06	31.10	33.72	30.90	33.76	29.29
B18	25.86	30.96	31.65	33.80	31.56	34.04	
B19	24.93	32.92	31.59	32.95	31.61	31.18	30.55
B20	24.23	28.98	28.73	28.20	29.50	30.60	29.14
B21	24.63	32.27	29.77	32.55	31.36	31.63	29.32
B22	24.66	29.06	31.47	30.38	32.16	31.38	29.20
B23	24.57	32.44	29.83	32.82	31.48	30.69	28.91
B24	24.15	32.23	28.76	28.47	30.19	31.14	31.55
B25	24.89	32.23	29.51	33.01	30.32	31.34	29.64
B26	25.08	32.52	32.10	33.11	32.68	30.74	31.63
B27	24.41	29.55	29.59	32.30	29.15	29.30	30.16
B28	24.01	29.12	28.82	31.56	27.73	28.75	28.84
B29	25.79	33.01	31.36	31.93	32.29	30.77	31.75
B30	24.35	29.35	29.56	29.78	29.04	29.29	29.36

Abbreviation: All samples were measured in triplicates, and the Cq values we provided are the mean value. The blank spaces were those Cq values more than 36.

Supplementary Table S3. The primer sequences of selected miRNAs in discovery phase for real-time PCR.

miRNAs	Sequences
miR-194-5p	UGUAACAGCAACUCCAUGUGGA
miR -204-5p	UUCCCUUUGUCAUCCUAUGCCU
miR -221-5p	ACCUGGCAUACAAUGUAGAUUU
miR -301a-3p	CAGUGCAAUAGUAUUGUCAAAGC
miR -30b-5p	UGUAAACAUCCUACACUCAGCU
miR -342-5p	AGGGGUGCUAUCUGUGAUUGA
miR -3605-5p	UGAGGAUGGAUAGCAAGGAAGCC
miR -4446-3p	CAGGGCUGGCAGUGACAUGGGU
miR -598-3p	UACGUCAUCGUUGUCAUCGUCA
miR -874-3p	CUGCCCUGGCCCGAGGGACCGA
miR -889-3p	UUAAUAUCGGACAACCAUUGU
novel-mir-451	CCAGACUGUCAGAUGCUCACC
miR-574-5p	UGAGUGUGUGUGUGUGAGUGUGU
novel-mir-67	CCAGGAUCGUUGACUCUGUGGCUC
novel-mir-9	CCAUGUGACUGAGACUCUGU

Supplementary Table S4. Mean Cq values of miRNAs in qRT-PCR in validation phase.

Sample	cel-miR-39	miR-301a-3p	miR-30b-5p	miR-342-5p	miR-4446-3p	miR-194-5p	novel-mir-67
C1	24.49	27.82	29.94	30.70	33.30	30.60	32.12
C2	25.45	29.03		27.86	30.70	27.96	30.88
C3	25.10	27.53	34.34	31.65	30.86	32.57	33.34
C4	25.12	27.99	30.38	30.28	31.65	30.70	31.59
C5	25.97	28.04	28.72	28.94	30.28	28.56	31.42
C6	25.87	28.80	31.34	30.32	28.94	31.17	31.60
C7	25.99	28.08	30.71	29.50	30.32	30.03	32.08
C8	25.98	27.85	28.18	31.65	29.50	28.56	30.13
C9	25.89	28.69	30.78	31.60	31.65	29.88	32.34
C10	25.68	29.12	28.49	30.09	31.60	29.18	31.77
C11	25.35	29.21	28.14	28.13	34.31	28.52	34.79
C12	25.56	27.65	29.32	29.39	32.09	29.62	34.71
C13	25.72	27.61	30.41	27.66	33.33	29.81	33.50
C14	25.96	27.28	28.96	30.78	28.89	28.03	33.77
C15	25.79	30.12	31.14	34.37	34.22	29.53	33.35
C16	25.89	28.2	28.03	29.32		27.89	
C17	25.52	27.87	28.88	31.53	30.13	30.57	34.41
C18	24.88	27.53	31.37	30.95	29.39	31.68	33.38
C19	25.57	29.00	32.86	27.65	29.66	29.13	35.12
C20	25.89	28.00	29.89		30.78	29.08	32.49
C21	25.52	27.56	28.49	29.42	34.37		34.25
C22	24.88	27.97	31.23	32.25	31.32	30.25	35.72
C23	25.57	28.86	29.20	27.35	34.61	28.99	34.61
C24	25.99	28.93	31.88	32.34	29.42	29.55	34.57
C25	25.99	27.54	28.05	28.09	32.25	30.28	33.85
C26	25.89	28.88	28.43	32.01	30.02	33.25	32.67
C27	25.89	28.92		28.30	32.34	28.03	
C28	25.10	27.63	30.07	30.32	32.09	30.02	34.67
C29	25.62	27.94	30.56	31.16	32.01	29.86	34.03
C30	25.97	28.17	34.02	29.42	28.97	28.22	32.97
C31	25.99	28.46	29.91	28.59	32.87		33.50
C32	25.99	28.13	34.47	29.65	32.13		33.00
C33	24.28	27.70	28.81	32.97	30.03	33.34	34.95
C34	25.42	29.48	30.01	28.79	32.84		34.01
C35	25.62	28.41	29.97	28.42	31.59	30.18	33.61
C36	24.08	27.79	32.17	31.17	29.65	29.95	33.49
C37	25.32	28.35	31.59	32.27	32.97	29.78	33.86
C38	24.30	29.17	32.91	29.41	28.79	31.51	34.06
C39	24.11	28.80	29.37	30.89	31.42	33.00	34.23
C40	24.09	29.28	29.40	29.31	31.17	28.23	34.98
C41	24.22	28.67	33.71	34.34	32.27	32.74	34.48
C42	24.13	27.53	30.82	29.90	29.41	28.27	31.81

C43	24.03	27.97	27.49	30.93	30.89	30.55	35.13
C44	25.79	31.61	31.42	32.36	32.42	30.66	33.00
C45	24.28	28.59	28.77	29.66	32.54	31.39	32.02
C46	25.82	29.89	33.34	31.80	30.78	28.00	30.72
C47	25.65	28.46	31.02	30.45	32.85	28.88	34.72
C48	25.55	27.63	29.77	31.79	29.56	30.09	34.01
C49	25.67	27.55	27.42	33.82	31.35	31.35	30.57
C50	25.66	27.61	28.61	27.45	32.48	29.12	33.40
C51	25.57	28.95	32.26	28.87	33.32	28.31	34.04
C52	25.36	29.34	29.66	30.91	32.83	28.50	34.68
C53	25.04	27.77	28.54	29.45	32.48	30.07	32.82
C54	25.24	27.94	33.04	30.59	29.00	29.94	34.66
C55	25.40	28.75	28.98	32.03	30.77	31.13	33.15
C56	25.64	30.42	30.79	33.17	31.61	28.15	32.02
C57	24.09	28.00	27.85	29.43	34.01	30.23	
C58	24.93	28.03	32.17	30.08	30.96	28.75	33.13
C59	25.02	28.78	29.23	28.92	30.59	28.13	33.32
C60	24.52	28.50	31.29	30.80	32.61	30.04	33.34
C61	24.07	27.63	29.93	32.43	30.22	28.53	31.51
C62	24.55	27.79	28.68	28.99	29.46	27.94	33.80
C63	24.38	27.94	27.84	31.58	29.52	30.62	33.49
C64	24.29	28.02	28.00	30.78	30.06	28.84	34.98
C65	24.40	28.13	28.85	29.86	29.20	30.80	30.72
C66	24.39	27.55	28.08	30.99	30.03	30.43	34.01
C67	25.08	27.97	30.75	32.52	28.79	31.19	34.68
C68	25.18	27.71	28.75	30.82	29.56	33.96	32.82
C69	25.21	28.24	28.67	30.86	29.79	28.71	32.02
C70	25.12	27.47	27.70	31.65	31.03	31.00	33.32
C71	25.14	27.07	29.48	30.28	29.68	29.87	31.51
C72	24.71	27.92	28.41	28.94	30.21	28.32	33.80
C73	25.25	28.05	27.79	30.32	28.87	34.81	32.70
C74	25.23	27.61	27.75	29.50	30.91	30.69	31.68
C75	24.91	28.29	30.99	31.80	29.45	31.43	30.39
C76	25.03	27.58	27.93	31.79	30.43	29.97	32.56
C77	25.64	28.20	30.50	28.87	28.74	28.39	34.92
C78	24.38	27.59	28.40	29.45	30.39	31.06	34.56
C79	25.18	30.07	30.12	33.80	30.02	30.68	33.15
C80	25.69	29.91	30.60	30.78	29.86	28.96	32.54
C81	24.58	28.81	28.01	28.94	31.72	29.00	33.77
C82	24.15	28.05	30.04	28.75	29.58	31.71	32.57
C83	25.69	27.53	31.39	30.77	30.15	30.92	33.25
C84	25.08	28.49	27.70	29.72	28.74	28.31	30.02
C85	24.35	27.56	27.53	27.93	28.93	31.03	29.86
V-A1	24.44	30.52	28.35	28.70	32.95	28.97	32.38

V-A2	25.56	29.57	30.26	29.89	30.10		34.76
V-A3	25.98	28.06	30.92	31.89	32.39	29.96	30.99
V-A4	25.89	30.67	28.40	30.23	29.95	29.62	31.98
V-A5	25.79	27.69	29.93	29.53	28.35	29.88	33.37
V-A6	25.90	29.40	29.44	29.94	31.29	29.91	
V-A7	25.80	28.34	30.20	29.97	30.62	29.96	32.86
V-A8	25.78	30.13	29.94	31.52	29.58	29.78	33.42
V-A9	25.69	28.05	30.43	31.00	29.46	28.21	29.92
V-A10	25.06	27.09	29.91	29.41	28.47	30.02	31.93
V-A11	24.14	29.16	30.46	30.18	32.14	30.42	32.22
V-A12	24.17	28.40	29.28	28.42	31.30	27.98	30.83
V-A13	24.35	29.95	31.08	30.02	29.48	30.29	31.54
V-A14	24.12	27.57	27.79	28.99	30.55	30.05	31.59
V-A15	24.03	29.01	30.22	29.07	31.22	29.36	31.42
V-A16	24.29	29.54	29.33	27.76	28.53	31.75	33.75
V-A17	24.31	27.71	27.93	30.38	31.95	28.49	31.97
V-A18	24.73	28.19	29.28	29.93	29.80	32.47	32.82
V-A19	24.84	28.56	28.67	29.05	30.63	30.00	31.03
V-A20	25.98	29.34	32.30	30.21	31.14	29.78	
V-A21	25.86	29.64	30.40	30.19	31.26	29.33	31.82
V-A22	25.97	28.99	32.74	32.32	29.94	29.56	34.58
V-A23	25.98	27.49	32.07	29.96	30.26	28.72	31.18
V-A24	25.83	28.09	29.70	30.31	30.37	29.48	32.98
V-A25	25.92	30.47	30.16	30.49	29.55	32.36	32.43
V-A26	25.76	28.01	30.19	29.79	31.51	28.82	31.78
V-A27	25.56	28.33	29.68	31.33	30.73	29.08	
V-A28	25.68	27.74	29.48	30.49	28.93	29.16	32.59
V-A29	24.72	28.70	31.04	29.36	29.92	28.09	31.61
V-A30	25.07	29.56	31.30	29.29	31.76	29.89	30.21
V-A31	24.14	28.57	30.67	30.75	30.15	30.46	32.15
V-A32	25.07	27.87	31.21	31.23	30.06	28.63	29.79
V-A33	25.06	28.12	30.57	29.31	31.91	29.07	29.48
V-A34	24.95	28.25	29.73	28.96	29.86	29.27	28.87
V-A35	24.74	27.13	32.37	29.98	28.40	28.81	30.91
V-A36	25.24	27.22	29.31	30.20	32.06	31.34	29.45
V-A37	24.06	28.27	32.23	28.84	31.04	31.21	30.59
V-A38	25.47	29.03	31.33	29.34	30.62	30.86	32.03
V-A39	24.26	29.54	28.41	29.86	29.30	31.27	31.17
V-A40	24.59	29.20	28.27	31.08	30.81	29.46	30.43
V-A41	24.29	27.32	29.20	29.90	31.09	29.22	29.68
V-A42	24.15	27.84	29.04	29.06	30.42	29.32	30.23
V-A43	25.79	29.65		28.56	30.14	28.75	30.03
V-A44	25.58	27.72	29.68	29.08	32.64	28.72	29.74
V-A45	25.86	30.30	30.54	30.65	31.98	31.43	28.98

V-A46	24.93		30.94	29.51	30.47	30.01	30.65
V-A47	24.23	27.62	29.99	29.56	32.27	29.25	29.94
V-A48	24.63	27.49	29.35	31.46	29.16	28.87	29.72
V-A49	24.66	28.08	28.28	30.15	29.67	30.27	29.57
V-A50	24.57	27.46	28.29	30.66	32.20	29.45	29.37
V-A51	24.15	29.06	29.09	29.17	30.75	29.86	29.79
V-A52	24.89	27.61	30.41	30.22	29.98	31.80	29.78
V-A53	25.08	28.29	31.50	28.52	30.51	32.33	31.58
V-A54	24.41	28.05	29.28	28.93	29.63	33.21	30.78
V-A55	24.01	28.40	30.50	28.82	30.81	28.68	29.86
V-A56	25.79	27.33	29.72	29.49	31.03	28.33	30.99
V-A57	24.35	27.35	29.12	29.38	30.12	28.35	30.04
V-A58	24.35	28.48	28.60	29.07	29.72	29.88	31.82
V-A59	24.90	28.97	32.05	28.88	31.96	30.46	30.93
V-A60	24.07	28.59	29.18	29.04	30.53	28.87	
V-A61	25.01	28.13	30.82	30.89	30.10	29.65	32.52
V-A62	24.31	30.52	29.39	28.94	29.58	28.07	33.17
V-A63	24.44	29.77	30.89	28.02	28.47	29.71	33.15
V-A64	25.56	28.75	28.11	29.29	31.14	27.98	29.00
V-A65	24.14	28.77	30.89	31.14	29.94	28.79	31.75
V-A66	24.03	28.27	28.94	28.79	30.37	30.21	31.82
V-A67	24.84	29.61	28.80	29.15	28.93	28.10	32.34
V-A68	25.98	28.88	29.48	31.17	31.76	32.03	30.91
V-A69	24.72	30.62	31.04	30.14	30.15	29.86	33.17
V-A70	25.87	29.17	31.30	30.50	32.03	28.22	30.49
V-A71	24.11	28.35	29.77	29.86	28.74	28.56	33.15
V-A72	24.29	30.50	30.77	30.11	30.72	29.33	31.35
V-A73	24.68	28.82	30.97	29.18	29.86	28.31	30.07
V-A74	24.94	27.14	28.40	28.93	29.37	29.02	33.21
V-A75	25.86	29.04	29.15	30.85	28.93	28.32	31.75
V-A76	24.14	27.93	27.93	29.16	28.94	31.85	31.23
V-A77	25.47	30.17	31.38	30.72	29.41	27.77	30.79
V-A78	24.22	32.72	30.64	28.68	30.89	28.13	33.17
V-A79	25.91	31.15	30.89	29.58	28.59	29.17	32.66
V-A80	25.72	30.33	29.38	29.81	30.96	28.73	31.17
V-A81	24.27	30.59	27.14	31.21	31.04	28.94	31.73
V-B1	25.79	29.39	30.30	29.15	31.17	31.60	33.65
V-B2	25.89	31.88	30.67	30.02	32.24	32.80	32.78
V-B3	25.78	33.3	31.21	30.77	30.59	32.94	
V-B4	25.91	30.93	32.57	28.97	30.90	31.82	30.44
V-B5	24.92	30.05	30.73	31.55	31.23	33.07	31.47
V-B6	25.24	34.25		30.71	32.61	30.81	30.71
V-B7	24.05	29.12	29.31	30.25	32.21	30.90	31.41
V-B8	24.10	33.07	31.33	30.04	32.00		31.67

V-B9	25.05	31.27	30.71	30.09	32.50	31.56	34.71
V-B10	24.88	31.64	30.17	30.88	33.02	29.89	
V-B11	25.19	32.30	31.25	32.75	32.19	30.26	33.92
V-B12	24.24	29.87	28.80	29.27	30.22	29.35	30.17
V-B13	25.72	31.63	31.89	31.26	30.85	29.85	33.70
V-B14	24.07	31.84	30.89	33.17	30.23	30.30	31.16
V-B15	25.47	31.22	30.23	31.15	31.10	31.97	31.61
V-B16	25.73	31.84	32.53	31.08	31.24	30.28	31.61
V-B17	24.32	33.25	28.94	30.73	31.16	30.95	30.13
V-B18	25.39	31.51	30.55	28.66	32.61	29.58	32.46
V-B19	25.10	32.56	32.48	30.52	29.46	30.22	30.25
V-B20	25.89	32.17	32.29	31.60	31.04	29.14	29.97
V-B21	24.27	32.59	33.80	29.49	31.63	29.51	31.33
V-B22	25.90	34.71	32.91	30.99	31.63		30.62
V-B23	24.03	33.30	30.37	32.51	30.84	30.63	33.06
V-B24	24.20	32.86	28.35	31.20	32.92	30.74	31.35
V-B25	25.79	31.16	31.23	29.56	31.31	33.23	34.21
V-B26	25.86	30.30	31.26	30.86	28.71	29.70	31.13
V-B27	24.28	35.05	29.49	30.71	30.71	29.93	30.48
V-B28	24.36	31.34	33.29	31.27	28.52	31.09	29.71
V-B29	25.78	30.19	30.66	28.41	30.06	29.11	31.61
V-B30	25.44	29.31	31.62	31.61	32.04	29.04	31.56
V-B31	25.71	30.67	31.44	30.79	32.01	31.29	32.00
V-B32	25.82	31.36	31.62	32.37	32.87	30.73	31.81
V-B33	25.85	31.86	30.29	31.13	32.29	30.07	32.64
V-B34	25.76	31.03	30.18	31.09	29.84	29.81	31.81
V-B35	25.78	31.88	31.40	30.52	30.13	29.55	32.67
V-B36	25.34	30.29	30.16	30.52	34.16	29.43	32.37
V-B37	25.89	32.54	28.43	33.32		31.62	32.14
V-B38	25.87	29.91	31.04	31.38	31.72	30.79	33.20
V-B39	25.54	31.13	30.47	30.75	31.58	30.63	31.10
V-B40	24.15	30.33	31.75	32.99	32.39	33.00	32.29
V-B41	24.22	31.94	32.27	29.97	32.46	32.07	31.61
V-B42	24.41	30.89	32.23	32.27	32.28	32.14	33.26
V-B43	25.78	30.76	28.59	29.35	33.04	31.05	33.17
V-B44	25.87	30.55	30.17	30.35	33.21	33.51	32.28
V-B45	25.37	30.82	32.97	30.73	33.21	30.13	32.26
V-B46	25.10	31.69	32.08	31.71	31.28	33.02	32.87
V-B47	24.97	29.53	29.00	30.30	31.40	29.78	32.86
V-B48	24.11	30.17	32.95	31.76	32.03	32.67	33.20
V-B49	24.57	31.84	30.75	32.53	31.75	29.43	32.18
V-B50	24.05	30.29	30.28	31.94	31.15	32.90	31.20
V-B51	24.29	29.64	29.35	31.07	32.02	33.11	33.31
V-B52	25.51	29.65	32.07	30.46	31.48	32.37	32.46

V-B53	25.69	32.05	30.06	32.63	30.69	32.47	32.64
V-B54	25.74	30.21	32.92	29.77	29.92	30.17	31.41
V-B55	25.25	29.84	32.58	28.75	29.77	30.14	31.19
V-B56	24.68	30.60	31.17	31.53	30.01	32.25	32.29
V-B57	24.58	30.24	30.46	29.72	29.97	30.03	31.16
V-B58	25.84	30.50	30.77	30.77	32.50	31.85	30.92
V-B59	25.85	29.40	30.97	30.97	30.85	32.24	34.37
V-B60	25.61	30.12	31.55	31.71	32.61	31.27	33.83
V-B61	25.90	33.88	30.99	32.27	28.71	31.78	34.39
V-B62	24.28	33.41	30.25	32.52	32.87	30.26	33.79
V-B63	25.71	34.97	29.74	29.65	34.16	31.23	30.37
V-B64	24.15	30.35	32.39	31.42	32.24	31.92	33.99
V-B65	25.10	31.30	29.72	30.37	32.61	33.19	
V-B66	24.05	33.09	30.77	29.72	32.00	28.95	29.75
V-B67	24.29	30.97	30.97	29.30	33.02	33.27	30.71
V-B68	25.69	30.06	32.71	32.53	31.63	29.69	29.89
V-B69	24.35	30.52	29.84	30.41	32.92	30.30	33.47
V-B70	24.73	29.61	30.08	31.80	30.06	31.75	30.45
V-B71	25.97	33.34	28.67	31.19	32.06	30.35	31.00
V-B72	25.76	31.61	30.80	32.31	33.30	33.36	33.62
V-B73	24.14	28.27	32.43	30.49	33.14	29.86	30.71
V-B74	25.10	31.17	30.32	33.56	32.20	31.27	29.11
V-B75	25.99	30.97	31.80	29.15	32.63	32.02	32.26
V-B76	25.42	33.71	31.79	31.53	30.69	32.26	30.16
V-B77	24.11	30.72	31.07	31.38	29.95	29.23	33.98

Abbreviation: All samples were measured in triplicates, and the Cq values we provided are the mean value. The blank spaces were those Cq values more than 36.

Supplementary Figure S1. A network of genes potentially targeted by five differentially expressed miRNAs.

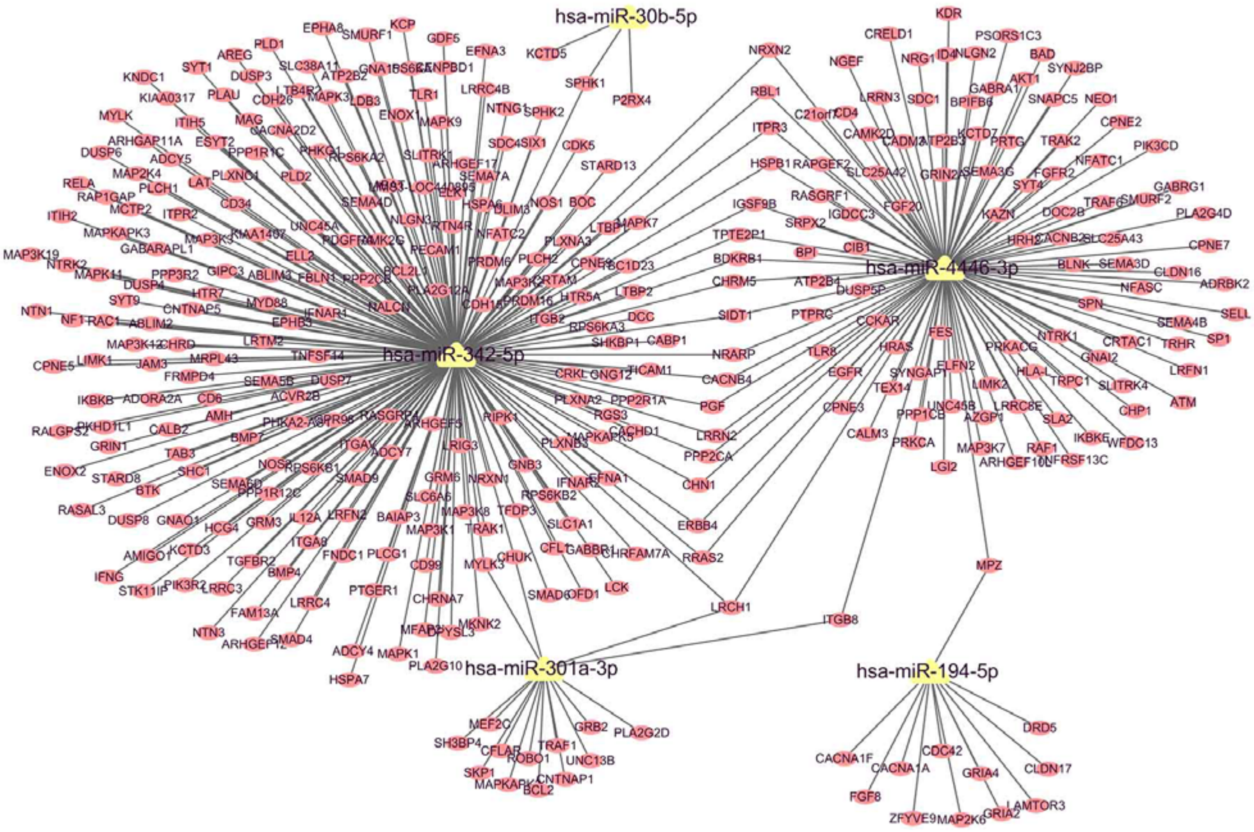

The yellow triangles represent the miRNAs, and the red circles represent the mRNAs of target genes. The gene symbol is presented as in NCBI at http://www.ncbi.nlm.nih.gov.
